# High-resolution genotype-free mapping of genetic variation with CRI-SPA-Map

**DOI:** 10.1101/gr.281514.125

**Published:** 2026-05

**Authors:** Sheila Lutz, Megan Lawler, Samuel Amidon, Frank W. Albert

**Affiliations:** Department of Genetics, Cell Biology, and Development, University of Minnesota, Minneapolis, Minnesota 55455, USA

## Abstract

Genetic variation within species shapes phenotypes, but identifying the specific genes and variants that cause phenotypic differences is costly and challenging. Here, we introduce CRI-SPA-Map, a genetic mapping strategy combining CRISPR-Cas9 genome engineering, selective ploidy ablation (SPA), and high-throughput phenotyping for precise genetic mapping with or without genotyping in the yeast *Saccharomyces cerevisiae*. In CRI-SPA-Map, a donor strain carrying SPA machinery is mated to a genetically different recipient strain harboring a genome-integrated selectable cassette. In the resulting diploid, CRISPR-Cas9 cuts the cassette for replacement with DNA from the homologous donor chromosome. Donor chromosomes are then removed using SPA to yield haploid recombinant strains. To establish CRI-SPA-Map, we mate a W303 SPA strain to 92 strains from the BY4742 yeast knockout collection that carry gene deletion cassettes on the left arm of Chromosome XIV and create 1451 recombinant isolates. Whole-genome sequencing verifies that deletion cassette replacement introduces short donor DNA tracts of variable length, resulting in a finely recombined mapping population. Using only the known locations of the gene deletions, which mark where donor DNA is introduced, we identify a 6.5 kb region shaping yeast growth. We further dissect this region and identify two causal variants in two genes, *MKT1* and *SAL1*. Engineering these variants alone and in combination reveals gene-by-environment interactions at both genes, as well as epistatic interactions between them that are dependent on the environment. CRI-SPA-Map is a cost-effective, meiosis-free strategy for creating high-resolution recombinant panels of yeast strains for identifying the genetic basis of phenotypic variation.

Most phenotypic traits, including morphological, physiological, and molecular quantities as well as susceptibility to common disease, vary continuously among individuals in a population. Studies in numerous species have shown that quantitative traits have a complex genetic basis, in which they are shaped by DNA variants at dozens to thousands of genes (Mackay and Anholt 2024). Each causal variant typically has a small effect on the trait, but because of the large number of causal variants, they contribute substantially to trait variation.

Two main experimental designs are used to dissect genetic variation in complex traits. Genetic mapping by linkage analysis involves crossing genetically different individuals to generate recombinant progeny, which are subsequently phenotyped for the trait of interest (Lynch and Walsh 1998). Genomic regions harboring causal alleles are identified through linkage with genetic markers determined by genome sequencing or other methods. Linkage analysis in crosses of two inbred parents can detect the effects of all variants that differ between the parents with equal statistical power, irrespective of their frequency in the wider population. However, the size of the regions identified by linkage mapping depends on the frequency of meiotic recombination and the number of recombinant progeny that can be both phenotyped and genotyped. Genome-wide association studies (GWASs) rely on past recombination events that have reshuffled genetic variation in natural populations of unrelated individuals (Visscher et al. 2012). GWASs can assay more variation than biparental crosses but have low power for variants that are rare in the population. GWASs are also prone to confounding by population structure (Peter et al. 2022) and by nongenetic, environmental factors (Young et al. 2019). The regions identified by linkage mapping and GWASs typically contain multiple variants and genes, complicating the identification of causal variants and mechanisms (Lappalainen et al. 2024; Mackay and Anholt 2024). Crucially, both linkage mapping and GWASs require that all individuals in the study are genotyped. Together, these reasons render the identification of individual genes and variants whose variation shapes complex phenotypes challenging.

The yeast *Saccharomyces cerevisiae* is a key model for the dissection of genetically complex traits (Liti and Louis 2012). The uniformly high meiotic recombination rate along the genome coupled with the ease with which large panels of strains can be generated has led to powerful applications of linkage analysis. Prominent efforts have included panels of thousands of progeny from a biparental cross (Bloom et al. 2015; Nguyen Ba et al. 2022); crosses that spanned multiple generations for additional recombination (Parts et al. 2011; Cubillos et al. 2013; Ziv et al. 2017; She and Jarosz 2018); the use of large pools of single, recombined cells (Ehrenreich et al. 2010; Parts et al. 2011; Albert et al. 2014); and crosses among multiple parents that cover much of the natural genetic variation in this species (Treusch et al. 2015; Bloom et al. 2019; Tsouris et al. 2024). Successful GWASs have also been conducted (Diao and Chen 2012; Skelly et al. 2013; Kita et al. 2017; Peter et al. 2018). However, applications of these approaches in yeast still suffer from many of the same shortcomings as in other species. In particular, the identified regions remain wide because of the limited ability of meiotic recombination to separate neighboring variants in even the largest panels.

New experimental designs for the dissection of complex traits have been developed in yeast. Reciprocal hemizygosity scanning in diploid hybrids can identify causal genes (Steinmetz et al. 2002; Weiss et al. 2018) but can be prone to false positives (Wilkening et al. 2014). In addition, when performed in a pool, unequal representation of cells that are hemizygous for each gene can lead to missed causal genes owing to differences in statistical power among genes. Quantitative noncomplementation screens have been used to pinpoint alleles with large effects but can also suffer from high false-positive rates (Kim et al. 2012). CRISPR-Cas9 genome engineering enables targeted introduction of genetic variants into genomes (DiCarlo et al. 2013). The high efficiency of homology-directed double-strand break repair in yeast has enabled massively parallel, pooled CRISPR-Cas9 strategies that can edit thousands of single variants in single cells. These methods make use of barcodes, guide RNA (gRNA) sequences, or repair templates as indicators of the intended edits in each cell (Bao et al. 2018; Roy et al. 2018; Sadhu et al. 2018; Sharon et al. 2018; Chen et al. 2023). However, not all genetic variants can be targeted by CRISPR, and not all regions in the genome have equal editing efficiencies. Because the targeted sites are not sequenced, there is no guarantee that any given cell in the pool is correctly edited. Genome-wide CRISPR engineering also requires synthesis of large libraries of gRNAs and repair templates. Beyond direct variant editing, CRISPR-Cas9 has been used to reshuffle yeast genomes by inducing mitotic recombination in diploid hybrids (Sadhu et al. 2016). However, this approach requires allele-specific gRNAs for the creation of DNA breaks in only one of the two genomes in the hybrid, which may not be possible in all genome regions. Despite these technical problems, yeast studies have revealed considerable complexity in the genetic basis of quantitative traits even within individual mapped regions, several of which have been found to harbor multiple causal genes or variants (Steinmetz et al. 2002; Gerke et al. 2009; Lutz et al. 2022; Schell et al. 2022).

A thorough understanding of the genetic basis of complex traits in *S. cerevisiae* and other species remains a fundamental challenge that requires new approaches. Here, we introduce CRI-SPA-Map, a new strategy for the systematic dissection of complex traits. CRI-SPA-Map combines CRISPR-Swap genome engineering (Lutz et al. 2019) in diploid hybrid strains of *S. cerevisiae* with selective ploidy ablation (SPA) (Reid et al. 2011). This engineering approach generates collections of haploid isolates with introduced variants that finely tile across the genome or a specific region of interest without relying on meiotic recombination. The known location of the introduced variants makes genotyping of these isolates unnecessary for genetic mapping. Additionally, CRI-SPA-Map provides improvements in spatial resolution over traditional mapping approaches, enabling identification of causal genes that shape complex traits.

## Results

### The CRI-SPA-Map strategy for transferring alleles between yeast strains

**Figure 1.**
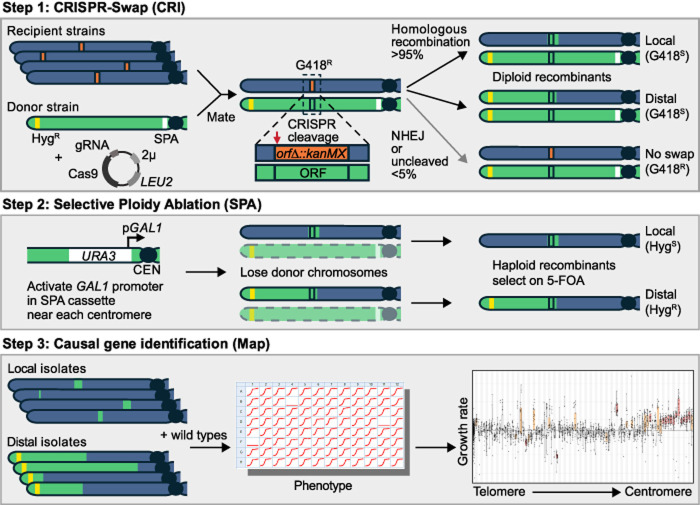
The CRI-SPA-Map procedure. For details, see text.

We developed CRI-SPA-Map, a genome engineering strategy that efficiently replaces alleles of a recipient strain with those from a donor strain at defined positions and allows genetic mapping with or without sequencing (Fig. 1). CRI-SPA-Map combines three key steps: (1) mating of a haploid recipient strain carrying a selectable marker cassette (e.g., KanMX) at a known genome position and a haploid donor strain to form a diploid hybrid, followed by replacement of the cassette with donor alleles via CRISPR-Swap (“CRI”) (Lutz et al. 2019); (2) removing the donor strain chromosomes through SPA (Reid et al. 2008, 2011) to create haploid isolates; and (3) mapping of causal alleles by associating phenotype with the introduced donor alleles in a collection of isolates. A related method, “CRI-SPA,” also mates a donor SPA strain to recipient strains but differs conceptually in that it drives a reporter construct from the donor into the same genomic location in each recipient (Cachera et al. 2023). In contrast, the final product of CRI-SPA-Map engineering is a collection of isolates in which selectable marker cassettes have been replaced with donor alleles.

To implement and test CRI-SPA-Map, we used the BY4742 (BY) *S. cerevisiae* gene knockout (YKO) collection as our recipient strains and a W303 strain as our donor strain. Each of the YKO strains has the same BY MATα genetic background but a different open reading frame (ORF) replaced with a KanMX cassette that confers resistance to the antibiotic G418 (Winzeler et al. 1999; Giaever et al. 2002). The genomes of W303 and the reference strain S288C, which is nearly identical to BY, differ at approximately 9476 single-nucleotide variants (SNVs), including nonsynonymous SNVs at approximately 700 genes (Matheson et al. 2017). The modest genetic divergence of these strains narrows the search space for dissecting quantitative traits and provides an excellent test bed for CRI-SPA-Map.

We focused on the left arm of Chromosome XIV (ChrXIV-L), which carries the known causal variant *MKT1*-D30G, which influences several complex traits including growth at high temperature, mitochondrial stability, and gene expression (Steinmetz et al. 2002; Deutschbauer and Davis 2005; Sinha et al. 2006; Demogines et al. 2008; Zhu et al. 2008; Dimitrov et al. 2009). We aligned BY and W303 sequences of ChrXIV-L and identified 445 SNVs and indels. We selected 92 BY YKO strains in which the KanMX cassette either replaced or neighbored these variants (Supplemental Table S1). For the W303 donor strain, we used a W303-SPA strain that is engineered to allow conditional loss of all W303 chromosomes (Reid et al. 2011). To the W303-SPA strain, we added a modified hygromycin-resistance cassette (HphNT1Δlinker) near the ChrXIV-L telomere and transformed this strain with the CRISPR-Swap plasmid (Lutz et al. 2019). This plasmid encodes a constitutively expressed Cas9 protein and the CRISPR-Swap gRNA that directs Cas9 to create a double-strand break within a linker sequence at the 5′-end of the KanMX cassette (Supplemental Fig. S1).

We mated the BY MATα YKO strains to the W303-SPA MATa strain and selected for diploids harboring the CRISPR-Swap plasmid (Fig. 1, step 1). In the diploid, the CRISPR-Swap Cas9/gRNA targets the KanMX cassette located on the BY chromosome and creates a double-strand break. Repair of this break using the W303 donor chromosome as a template removes the KanMX cassette and renders the cell sensitive to G418 (G418^S^). Rarely, cells are repaired by nonhomologous end joining (NHEJ) or have no repair. Such cells are easily identified as they remain resistant to G418 (G418^R^). Importantly, it can be assumed that G418^S^ cells have repairs that reinstate the deleted ORF with the W303 allele.

Replacement of the KanMX cassette with W303 alleles can occur by different double-strand break repair mechanisms to create “local” or “distal” incorporation of W303 alleles (Yin and Petes 2013). Local repairs are products of homologous repair by gene conversion, which typically incorporates variants only near the site of repair at the ORF and its surroundings. Distal repairs arise from break-induced repair, which incorporates variants from the centromeric side of the ORF to the telomere (Fig. 1, step 1).

We induced SPA of the W303 chromosomes by activating conditional *GAL1* promoters at each centromere (Fig. 1, step 2). Transcription from the *GAL1* promoters leads to centromere instability and loss of the W303 chromosomes during mitotic division (Reid et al. 2008). We selected cells that lost all W303 chromosomes via growth on galactose and 5-FOA, a compound that is toxic to cells expressing the *URA3* gene upstream of the *GAL1* promoter at each W303-SPA centromere.

In haploid cells after SPA, the local and distal repair types can be distinguished by selection on hygromycin (Fig. 1, step 2). Cells with distal repairs are resistant to hygromycin (Hyg^R^) because of the introduced HphNT1Δlinker cassette near the ChrXIV-L telomere. In contrast, cells with local repairs do not introduce the cassette and are hygromycin sensitive (Hyg^S^).

Following SPA, we obtained an average of 171 colonies per targeted ORF (range of 42–305) (Supplemental Table S2). The colonies for one YKO strain, YNL084C, were slow to form owing to a *GAL2* mutation in this strain (see below). On average, 67% of colonies were Hyg^S^ based on replica plating to YPD + Hyg, consistent with having cells with a local repair. The remaining 33% of colonies contained Hyg^R^ cells, consistent with distal repairs. Seven of the targeted ORFs had a notably small fraction (<7%) of Hyg^R^ colonies, which we found was caused by engineering unexpected YKO strains (discussed below).

We single-colony-streaked Hyg^S^ and Hyg^R^ colonies from each YKO strain to ensure CRI-SPA-Map isolates with unique repair events. We assayed each isolate to verify the expected genotypic markers, including auxotrophic markers that distinguish BY from W303, loss of the KanMX cassette, loss of the CRISPR-Swap plasmid, and absence or presence of HphNT1 for local or distal repairs, respectively (Supplemental Table S2). In total, we obtained 1451 isolates for further analyses.

### CRI-SPA-Map transfers narrow tracts of DNA between yeast strains

**Figure 2.**
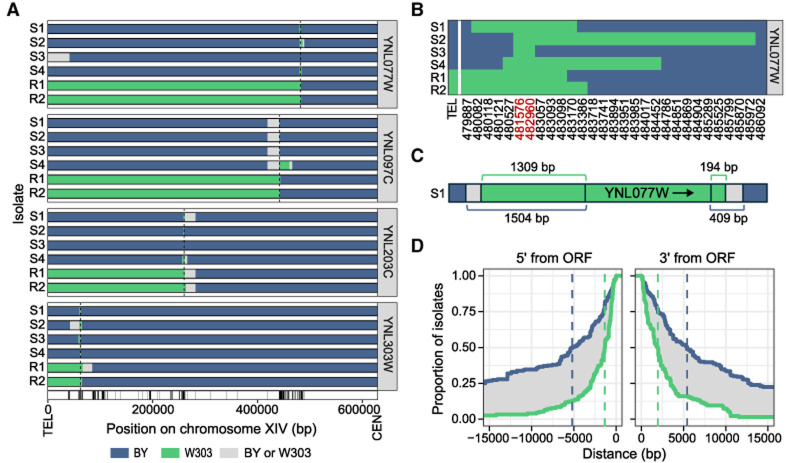
CRI-SPA-Map isolates contain tracks of W303 variants on ChrXIV-L. (*A*) Position of W303 tracts in four local (S for Hyg^S^) and two distal (R for Hyg^R^) isolates from four YKO strains. W303 tracts in all isolates are depicted in Supplemental File S1. BY alleles are in blue and W303 alleles in green. The sequence between the last W303 variant in the tract and the first BY variant could originate from either the BY or W303 strain and is therefore shown in gray. Targeted ORF positions are shown with dotted lines. Variant positions are marked by tick marks with the position of the telomere (TEL) and centromere (CEN) at the *bottom* of the plot. (*B*) Allele designation at each variant (not drawn to scale) in isolates of YNL077W. Variants within the ORF are in red. (*C*) The W303 variant tract at YNL077W ORF in isolate S1 (drawn to scale), with distances to the most distal W303 and nearest BY variants at each end of the ORF. (*D*) Cumulative distribution of 286 isolates from 69 YKO strains with the last W303 and first BY variant at the indicated distance from the 5′-end and 3′-end of the ORF. Vertical dashed lines designate the maximum (blue) and minimum (green) of the median tract lengths. For distal isolates, only the centromeric side was analyzed. Isolates with mosaic tracts or W303 tracts that do not extend beyond the ORF were excluded.

To explore the genomic outcomes of CRI-SPA-Map engineering, we whole-genome-sequenced 555 CRI-SPA-Map isolates (typically four local and two distal isolates) (Supplemental Table S2) created from the 92 YKO strains. We analyzed genotype calls at 8257 high-quality variants across the genome (7954 SNVs and 303 indels) (Supplemental Table S3). These analyses confirmed that CRI-SPA-Map successfully incorporated donor DNA from W303 into the recipient BY YKO strains on ChrXIV-L (Fig. 2A; Supplemental Fig. S2; Supplemental File S1).

In some isolates, we identified genome changes beyond, or instead of, the expected W303 variants on ChrXIV-L (Supplemental Table S2). In 12.3% (68/555) of sequenced isolates, these reflected preexisting genome alterations, well-to-well contamination, or mislabeling in our copy of the YKO collection. For example, all six sequenced isolates from the YKO strain of YNL079C/*TPM1* carried a duplication of ChrIX containing the paralog of this ORF, suggesting this aneuploidy predated CRI-SPA-Map engineering (Supplemental Fig. S3). Isolates from the seven YKO strains with a low fraction of Hyg^R^ colonies had genotypes indicating that the KanMX cassette was not located on ChrXIV-L. For example, all local isolates of YNL173W had W303 variant tracts at YMR316W on ChrXIII-R (Supplemental Fig. S4). When most sequenced isolates from a YKO strain showed evidence of shared unexpected genome alterations or targeting of the wrong ORF, we flagged all isolates from that strain as affected by issues in the YKO collection.

In 3.4% (19/555) of the sequenced isolates (Supplemental Fig. S4), we observed W303 variants on the right arm of Chromosome XII (ChrXII-R) that extended from the rDNA locus to the telomere. To determine the frequency of this recombination event, we screened the unsequenced isolates by utilizing a polymorphism in the *HAP1* gene located between the rDNA locus and the ChrXII-R telomere that confers resistance to fluconazole (Gaisne et al. 1999; Saha et al. 2024). In total, we identified 2.8% (40/1451) of isolates with a recombination on ChrXII-R (Supplemental Table S4).

Apart from rDNA recombination, only 2.9% (16/555) of the sequenced isolates from 13 YKO strains had unexpected genotypes that might be the result of CRI-SPA engineering. In six isolates, heterozygous genotypes surrounded the targeted ORF, suggesting these isolates are diploid. In another six isolates, W303 variants were located on a chromosome other than ChrXIV-L. Finally, five isolates were aneuploid, one of which also had heterozygous genotypes. Thus, CRI-SPA-Map-based engineering rarely caused unintended genome alterations.

We examined the W303 DNA tracts introduced into each isolate on ChrXIV-L (Fig. 2A; Supplemental Table S5) and found variation in tract length, even among isolates derived from the same YKO strain (Fig. 2B). In 3.2% (18/555) of the sequenced isolates, we observed alternating tracts of W303 and BY variants flanking the targeted ORF (Supplemental Fig. S5). These mosaic tracts have been previously observed as an outcome of double-strand break repair in diploids (Yin et al. 2017; Gorter de Vries et al. 2019).

To determine the length of the W303 tracts, we identified the most distal W303 allele and the nearest BY allele at each end of the targeted ORF. The repair tract end point lies between these two variants, with resolution limited by the density of W303/BY variants (for an example, see Fig. 2C). Across isolates, the donor tract length at each end of the ORF was between 1532 bp for the last W303 allele and 5397 bp for the first BY allele (Fig. 2D). Among the local isolates, even the larger of these two estimates added to the size of an average ORF corresponds to repair tracts of ∼12 kb, 10 times smaller than typical linkage blocks in meiotic crosses (e.g., 117 kb in the BY/RM cross) (Supplemental Methods; Bloom et al. 2013).

Most W303/BY variants are positioned close enough to a YKO ORF to be incorporated into typical repair tracts (92.8% and 99.99% for the two median length estimates). Similar proportions apply to the fourfold more variants in the widely used BY/RM strain pair (93.8% and 99.8%) (Supplemental Fig. S6). Thus, the vast majority of variants in yeast crosses are accessible to CRI-SPA engineering.

### Mitochondrial sequence varies among CRI-SPA-Map isolates

The mitochondrial sequence of the CRI-SPA-Map isolates, even from the same YKO strain, showed a variety of patterns of BY and W303 variants (Supplemental Fig. S7), indicative of both parental and recombinant mitochondria types (Berger and Yaffe 2000; Fritsch et al. 2014). To control for potential effects of mitochondrial genotype in our genetic mapping, we created 16 wild-type isolates using a BY4742 strain without a KanMX cassette as the recipient in the CRI-SPA-Map procedure. The sequence of six of these wild-type isolates also showed shuffling of the BY and W303 mitochondrial variants (Supplemental Fig. S7).

### CRI-SPA-Map identifies a narrow causal genomic region

To test the utility of CRI-SPA-Map isolates for mapping complex traits, we measured growth rates in liquid YPD medium of 1259 isolates (644 local and 615 distal) together with the 16 wild-type isolates (Supplemental Table S6). Typically, seven local and seven distal isolates for each of the 92 YKO strains were phenotyped. We analyzed the growth rate of 1057 isolates from 78 ORFs after excluding isolates with issues arising from the YKO collection or recombination on ChrXII-R.

To identify ORFs with or near causal variants, we grouped the CRI-SPA-Map isolates by their targeted ORF and compared their growth rates to the wild-type isolates. This strategy, which we term “ORF-based mapping,” leverages the fact that any donor variants within the targeted ORF are guaranteed to be introduced, and any additional donor variants will extend from this ORF. As such, ORF-based mapping utilizes the known locations of the targeted ORFs and does not require knowledge of the precise genotype of each isolate.

**Figure 3.**
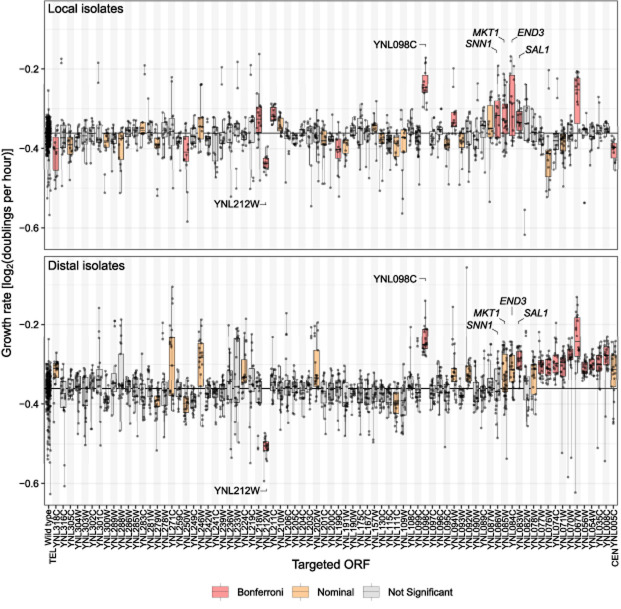
Growth rate in liquid YPD of local (*top*) and distal (*bottom*) isolates grouped by targeted ORF. Targeted ORFs are ordered by chromosomal position from telomere (TEL) to centromere (CEN). ORFs discussed in the text are labeled. Vertical lines connect replicate measurements (points) for each isolate. Box plots are based on mean measurements for each isolate; the central line marks the median, and the box extends from the first to third quartile. Growth rate is compared to the wild-type isolates on the far *left*. The median of the wild-type isolates is marked with a horizontal line through the plot. Red boxes indicate significance at the Bonferroni threshold of *P* < 0.00064; orange, *P* < 0.05. Phenotypes of 532 local and 525 distal isolates are shown.

The local isolates from 14 ORFs had growth rates significantly different from the wild-type isolates based on mixed linear models and a Bonferroni-corrected *P*-value threshold of *P* < 0.00064 (Supplemental Table S6). A cluster of significant genes occurred at *MKT1* and its three neighboring genes, *SNN1*, *END3*, and *SAL1*. These four genes all had effects in the same direction, with the introduced W303 allele causing an increased growth rate (Fig. 3, top).

Because of the compact nature of the yeast genome, in which most ORFs are separated by a few hundred bases of intergenic DNA, the introduced W303 DNA tracts typically comprise at least some of the genes neighboring a targeted ORF. Therefore, when several adjacent ORFs are targeted, we expect the effects of any causal variants to spill over into isolates of the neighboring ORFs, creating a wave of significance (Supplemental Fig. S8A–C). This pattern is seen at *SNN1*, *MKT1*, *END3*, and *SAL1*, but nowhere else along ChrXIV-L. The ORFs YNL098C and YNL212W had significant effects, whereas their neighboring ORFs had nonsignificant effects or did not show an effect in the same direction (Fig. 3, top).

To further investigate these observations, we analyzed the distal isolates (Fig. 3, bottom). To rule out the possibility that the HphNT1Δlinker cassette in distal isolates affects growth, we compared BY4742 strains with this cassette to the wild-type isolates and found that their growth rates were not significantly different (*P*-value = 0.47) (Supplemental Fig. S9).

Distal isolates carry tracts of W303 alleles extending from the targeted ORF to the telomere. A causal W303 allele is included in a distal isolate if the causal variant is located either in the targeted ORF or between the targeted ORF and the telomere (Supplemental Fig. S8A–C). The causal allele can also be contained in distal isolates from ORFs located telomeric but in close proximity to the causal variant. Growth rates of distal isolates plotted against the position of their targeted ORF, ordered from telomere to centromere, should change as they begin to incorporate the causal variant. This change will then be followed by a plateau of similar growth rates for ORFs on the centromeric side of the causal variant, because these isolates all contain the causal donor allele.

The growth rate of distal isolates began to climb for ORFs just telomeric of *MKT1* (first without reaching statistical significance, e.g., at *SNN1*), became different from the wild-type isolates at nominal significance for *MKT1* and *END3*, reached Bonferroni-significance for *SAL1*, and continued to be nominally significant for 12 (10 of which with Bonferroni-significance) of the remaining 13 targeted ORFs between *SAL1* and the centromere. All of these significant ORFs had estimated allele effects in the same direction (Fig. 3, bottom).

In contrast, no rise or fall followed by a plateau was seen at YNL098C and YNL212W, which once again stood out as prominent outliers (Fig. 3). Given the consistency in phenotypes from these two ORFs among the local and distal isolates and the unexpected specificity of their association signals compared with neighboring ORFs, the most parsimonious explanation is that the observed phenotypes are not caused by introduced W303 variants at these loci. Instead, they likely reflect preexisting variants in the YKO strains from which the isolates were derived (see below).

To examine the spatial resolution and statistical power of CRI-SPA-Map, we focused on the region of adjacent ORFs (*SNN1*, *MKT1*, *END3*, and *SAL1*; YNL086W to YNL083W) that had reached statistical significance in the local CRI-SPA-Map isolates. From the start of the *SNN1* ORF to the stop of the *SAL1* ORF, this region spans 6527 bp, which is about six times smaller than typical yeast growth QTLs (e.g., a median of ∼40 kb in the work of Nguyen Ba et al. 2022) and eight times smaller than typical bulk-segregant QTLs (e.g., medians of 54–68 kb in the work of Albert et al. 2014 and Collins et al. 2022). The average estimated fold increase in growth rate caused by the region demarcated by these four genes was 3% (Supplemental Table S6). Thus, CRI-SPA-Map has the ability to map genomic regions shaping quantitative traits with high spatial resolution and statistical power sufficient to detect subtle effect sizes typical of natural genetic variation.

### Incorporation of sequence data improves mapping resolution

We incorporated our WGS data to determine if knowledge of the specific W303 alleles introduced into each isolate augments the ORF-based mapping results. We tested each BY/W303 variant by comparing the phenotypes of CRI-SPA-Map isolates that carried the given W303 allele to those that carried the BY allele. These analyses grouped isolates based on their observed genotypes irrespective of which targeted ORF the isolates had been generated from. Because not all isolates had been sequenced, these analyses represent a trade-off between a smaller sample size and the additional precision gained from incorporating genome sequence information.

**Figure 4.**
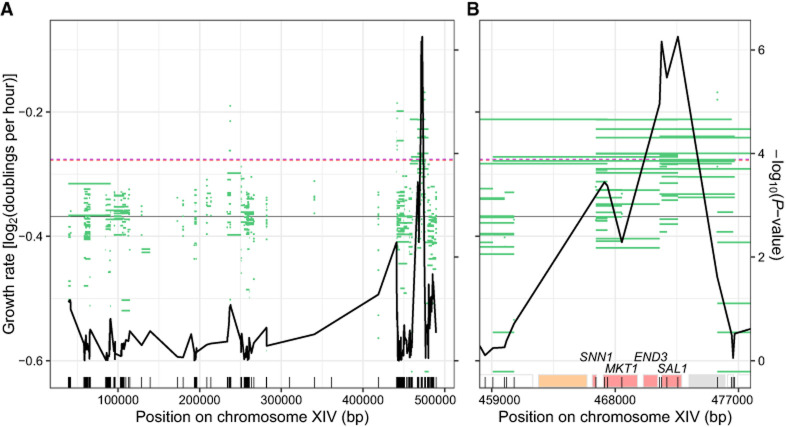
Incorporation of genotype data in CRI-SPA-Map for local isolates. (*A*) Variant positions on ChrXIV-L are depicted with vertical tick marks on the *bottom*. Green lines depict W303 allele tracts in each of 332 local isolates as well as isolate growth rates in liquid YPD. The solid black line shows the −log_10_(*P*-value) at each variant as indicated by the right *y*-axis in panel *B*. The median growth rate of all local isolates is denoted by a solid horizontal gray line. Dashed lines denote significance thresholds as determined by Bonferroni correction (red) and 1000 permutations (purple). Note that these thresholds were nearly identical. (*B*) Zoom-in on the region containing the four significant variants. The ORFs in this region are depicted as rectangles, with those used in ORF-based mapping colored based on the significance of their effect as in the *upper* panel of Figure 3.

In the local isolates, these analyses showed a narrow peak, at which four variants located within 1333 bp exceeded a permutation-based significance threshold of *P* < 0.000128 (Fig. 4A,B; Supplemental Table S7). The four variants were located inside or 135 bp upstream of the *SAL1* ORF. The most significant *P*-value was observed at the *sal1-1* allele, in which a 1 bp insertion in BY4742 causes a frameshift at codon 403 of 545 (Chen 2004). The *sal1-1* frameshift had many missing genotype calls in our initial sequence analysis and required manual curation (Methods). The distal isolates showed a wider peak extending from *MKT1* to *SAL1*, reflecting more extensive linkage in distal isolates (Supplemental Fig. S10A,B). Thus, incorporation of genotyping data for CRI-SPA-Map isolates can further increase mapping resolution. This is especially true for local isolates, in which repair tracts are short and vary on both sides of a targeted ORF.

### Variants in *MKT1* and *SAL1* shape growth in liquid YPD

**Figure 5.**
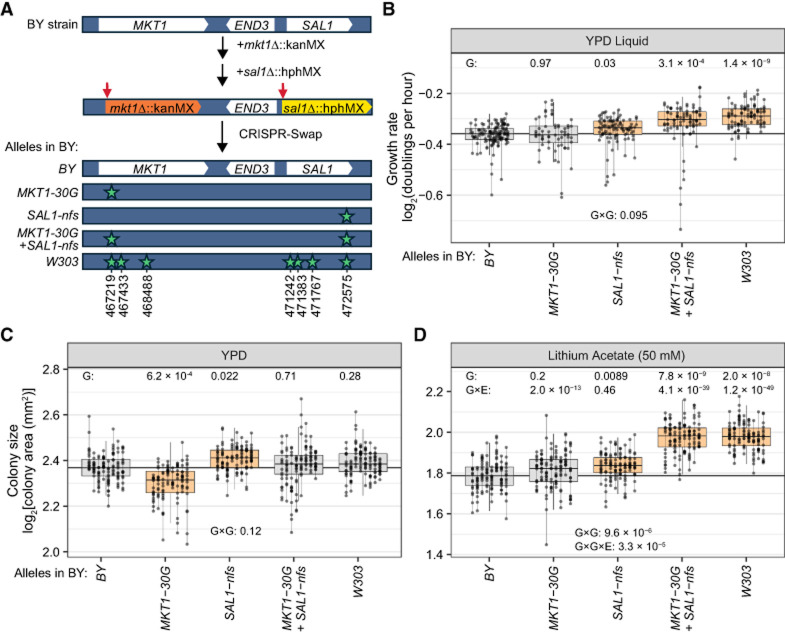
Effects of the *MKT1-30G* and *SAL1-nfs* alleles. (*A*) Engineering of the *MKT1*-*END3*-*SAL1* locus by double-cut CRISPR-Swap. In a BY strain, the *MKT1* and *SAL1* ORFs were replaced with KanMX and HphMX, respectively. These two cassettes were cut by CRISPR (red arrows), and the locus was replaced by the depicted alleles (variant positions are starred) provided on a PCR-amplified fragment. (*B*) Growth rates in liquid YPD. *P*-values are shown for comparing each genotype to the engineered BY control (G) and for the test of the epistatic interaction between the *MKT1-30G* and *SAL1-nfs* alleles (G×G). Box plots are orange if *P* < 0.05. Growth measurements from the same strain are connected by a vertical line. Box plots as in Figure 3. The median of the BY wild-type strain is depicted as a horizontal line through the plot. (*C*) Colony size of engineered strains after 40 h of growth on solid YPD. Box plots and *P*-values for G and G×G are as in *B*. (*D*) Colony sizes on solid YPD with lithium acetate. *P*-values for G×E interactions comparing the given condition to solid YPD are indicated for each genotype, along with that for the G×G×E term.

To independently validate and further dissect the region identified by CRI-SPA-Map, we used double-cut CRISPR-Swap (Lutz et al. 2019) to engineer the region from *MKT1* to *SAL1* in the BY background (Fig. 5A). We focused on this region because of the significance of *SAL1* in both local and distal ORF-based mapping and because the *sal1-1* frameshift is known to increase *petite* frequency and has been linked to slowed growth on ethanol (Dimitrov et al. 2009; Schell et al. 2022). Additionally, *MKT1*-D30G is a known causal variant for numerous traits (Fay 2013).

The engineered region harbors seven BY/W303 variants (Fig. 5A). BY strains with all seven W303 alleles had significantly higher growth rates in liquid YPD compared with strains in which all seven BY alleles were reintroduced (Fig. 5B), validating the discovery of this causal locus by CRI-SPA-Map. Next, we introduced the glycine-coding “G” allele at *MKT1*-D30G and the frameshift-free “*SAL1-nfs*” W303 alleles into BY, individually and in combination. BY strains harboring *SAL1-nfs* grew significantly faster than the control (*t*-test *P*-value = 0.03), whereas the *MKT1-30G* allele had no effect on growth (*P* = 0.97) (Fig. 5B).

Combining *MKT1-30G* and *SAL1-nfs* led to significantly faster growth relative to BY (Fig. 5B), at a rate not different from that of BY strains carrying the entire W303 region (*P* = 0.15). Therefore, these two variants are responsible for most, and perhaps all, of the effect of this region. BY strains with both alleles grew significantly faster than those with the *MKT1-30G* allele alone (*P* = 0.018), as expected given the effect *SAL1-nfs* had in isolation. Strains with the combined alleles also grew faster than strains carrying just the *SAL1-nfs* allele (*P* = 0.030), suggesting a contribution from *MKT1-30G* in the context of the *SAL1-nfs* allele even though it had no effect in isolation. Although a formal test for a genetic interaction between *MKT1-30G* and *SAL1-nfs* failed to reach statistical significance (ANOVA, interaction *P*-value = 0.095), these results suggest epistasis between these two alleles such that their joint effect exceeds that of their individual effects.

### Gene-by-gene, gene-by-environment, and gene-by-gene-by environment interactions of the *MKT1* and *SAL1* causal alleles

To explore the extent of epistasis between *MKT1-30G* and *SAL1-nfs* (i.e., G×G) and the environmental dependence of the effects of these alleles (i.e., G×E), we grew our strains carrying all combinations of these two alleles on 11 diverse solid media and measured colony size after 40 h (Supplemental Fig. S11; Supplemental Table S8). Colony size is often used as a proxy for the number of cell divisions (a quantity closely related to the growth rate); however, smaller colonies can also arise from smaller cells without a change in growth rate. The *SAL1-nfs* allele increased colony size on solid YPD (*t*-test *P*-value = 0.022), mirroring its effect on growth rate in liquid YPD. In contrast, the *MKT1-30G* allele significantly reduced colony size on solid YPD (*P* = 6.2 × 10^−4^), even though it had no effect on growth rate in liquid YPD (Fig. 5B,C). The combined *MKT1-30G* and *SAL1-nfs* alleles had no significant effect on solid YPD relative to BY (*P* = 0.71) and showed no deviation from additivity (G×G test: *P*-value = 0.12). This contrasts with their joint effect in liquid YPD, in which they significantly increased growth rate. These analyses demonstrate that colony size on solid YPD and growth rates in liquid YPD are distinct, noninterchangeable traits.

An epistatic relationship between the *MKT1-30G* and *SAL1-nfs* alleles was observed on lithium acetate (G×G test: *P* = 9.6 × 10^−6^) (Fig. 5D). This interaction involved no individual effect for *MKT1-30G*, a slight increase in colony size for *SAL1-nfs*, and a joint effect of these alleles that led to significantly larger colony sizes than the sum of their individual effects (Fig. 5D).

Compared to solid YPD, G×E was exhibited for *MKT1-30G* in nine and for *SAL1-nfs* in five of the 10 conditions (G×E: *P*-value < 0.05) (Supplemental Fig. S11; Supplemental Table S8). We observed no cases in which an allele had significant effects in both the tested condition and solid YPD that were in opposite directions. Specifically, *MKT1-30G* reduced colony size compared with the wild-type control in seven conditions (*P* < 0.05), with no significant effect in the remaining conditions. Conversely, the *SAL1-nfs* allele increased colony size in six conditions and had no significant effect in the remaining conditions.

The epistatic effect of *MKT1-30G* and *SAL1-nfs* seen on lithium acetate was not present on solid YPD, resulting in an environment-specific genetic interaction (G×G×E: *P*-value = 3.3 × 10^−5^) (Fig. 5D). Four additional conditions showed G×G×E (Supplemental Fig. S11). These conditions differed from solid YPD in that the joint effects of *MKT1-30G* and *SAL1-nfs* were more similar to those of *MKT1-30G* than to those of *SAL1-nfs*, even though none of these conditions showed significant epistasis (Supplemental Fig. S11). Together, these results show that the effects of causal alleles can depend strongly on the environment and that even small genomic regions can contain multiple causal variants.

### Strain-specific variants in the YKO collection

Strain-specific variants in the YKO collections have been reported to influence phenotypes (Lehner et al. 2007; Teng et al. 2013; Van Leeuwen et al. 2016). We therefore asked whether background variants are present in our CRI-SPA-Map isolates derived from the YKO collection. In total, we identified 86 background variants across 53 of 84 (63.1%) YKO strains. These variants are predicted to introduce four frameshifts, six premature stop codons, and 41 amino acid substitutions affecting 45 genes (Supplemental Table S9).

A variant introducing a frameshift in *GAL2* likely explains the slow growth of the YNL084C YKO strain on galactose we observed (Tschopp et al. 1986). We also identified one background variant in the YNL098C YKO isolates and three in the YNL212W YKO isolates, which may account for the large growth difference observed in these isolates relative to the wild type and most other YKO strains. Together, these findings indicate that background variants present in the YKO collections can influence phenotypes in CRI-SPA-Map isolates.

## Discussion

We have introduced CRI-SPA-Map, a new strategy for genetic mapping of complex traits. CRI-SPA-Map generates collections of otherwise genetically identical individuals with variants introduced from a donor strain at defined genomic regions. We applied CRI-SPA-Map to dissect genetic variation on a chromosome arm between a W303-SPA strain and strains from the YKO collection and uncovered a complex pattern of environmentally dependent epistasis at two causal variants.

CRI-SPA-Map has several key advantages. First, using CRI-SPA-Map to target an ORF that had been deleted with a selectable marker cassette guarantees that this ORF is replaced with donor DNA. This property enables mapping without genotyping, as the known position of the deleted ORF specifies the genomic location of the introduced donor DNA. Although whole-genome sequencing of individual yeast strains is now routine and affordable, the materials and labor cost of constructing sequencing libraries for hundreds of strains remains a significant hurdle. Additionally, CRI-SPA-Map enables mapping in regions that are hard to genotype, such as repetitive regions of the genome. Second, CRI-SPA-Map has high resolution owing to the short introduced donor DNA tracts in local isolates and tracts that terminate near the targeted ORF in distal isolates. Resolution can be further increased by targeting stretches of neighboring ORFs, in which partially overlapping repair tracts of variable length create a panel of isolates with densely interlocking recombination points. Third, statistical power is equally high for all variants between the donor and recipient, unlike designs in which allele frequencies vary naturally as in GWASs or from inadvertent selection and drift in advanced intercrosses (Ziv et al. 2017). Lastly, the cost of creating panels of CRI-SPA-Map isolates is minuscule, as only yeast media, plates, and equipment for handling and phenotyping isolates are needed.

We verified the fidelity of CRI-SPA-Map engineering by whole-genome-sequencing 555 CRI-SPA-Map isolates. Tracts of W303 variants were integrated at the targeted KanMX cassette at each ORF in the YKO collection, and CRI-SPA-Map introduced few off-target genome alterations.

Using local isolates for ORF-based mapping, we identified a 6.5 kb genomic region including four genes, *SNN1*, *MKT1*, *END3*, and *SAL1*, that caused a growth difference in liquid YPD. Mapping with distal isolates suggested *SAL1* as the causal gene. Sequencing corroborated these results and further increased mapping resolution. Genome engineering confirmed that a frameshift in *SAL1* (*sal1-1*) caused this growth difference. Although the *MKT1*-D30G variant affects numerous complex traits (a reason we opted to study ChrXIV-L), it had no significant effect on growth rate in liquid YPD. Identification of this region, and particularly the single gene *SAL1*, demonstrates the effectiveness of CRI-SPA-Map at high-resolution genetic mapping of complex traits.

Further work revealed a gene-by-gene (G×G) interaction between the two alleles, gene-by-environment interactions (G×E) for each of the alleles, and gene-by-gene interactions between the alleles that are dependent on the environment (G×G×E). These results highlight the complexity of variant effects even within a small genomic region.

The mechanism behind these condition-specific genetic effects remains unclear but may involve the formation of *petites*, yeast cells lacking functional mitochondrial DNA. Both the ancestral *MKT1-30G* allele in W303 and the derived *sal1-1* allele in BY increase the rate of petite formation, likely through different mechanisms (Dimitrov et al. 2009). Mkt1, an RNA-binding protein, possibly targets mRNAs to the mitochondrion (Lee et al. 2009; Caballero et al. 2025), whereas Sal1, an ADP/ATP transporter, helps maintain mitochondrial ATP levels and inner membrane potential (Traba et al. 2008). Introduction of the *MKT1-30G* allele into BY led to decreased colony size in multiple conditions, possibly owing to a higher proportion of *petite* cells. Conversely, introduction of the W303 *SAL1-nfs* allele into BY increased colony size in multiple conditions, possibly by decreasing petite formation.

Although CRI-SPA-Map is effective and efficient at mapping genetic variation, limitations do remain. Most prominently, CRI-SPA-Map relies on compatible pairs of donor and recipient strains. Here, we opted for the W303/BY4742 pair because a SPA strain and the YKO collection exist in these backgrounds. These strains are also largely syntenous. In more distantly related strains, structural rearrangements may create incompatibilities following double-strand break repair. More generally, engineering in repetitive regions may cause unintended consequences. For example, a double-strand break between two direct repeats might render cells G418^S^ without the introduction of donor DNA.

A shortcoming of the W303/BY strains arises from their close genetic relatedness, especially because W303 was created in part by crossing BY to other strains (Matheson et al. 2017). This history has resulted in unusual patterns of variation, including clusters of variants separated by long stretches without variants. This limited our ability to precisely pinpoint DNA repair breakpoints. The low overall amount of variation also limits the number of causal variants and may not be representative of other strain pairs.

Our current implementation of CRI-SPA-Map relies on SPA to remove the donor genome. To our knowledge, W303 is the only strain with SPA cassettes at all centromeres, enabling complete donor genome removal. Additional SPA strains could be generated as previously described (Reid et al. 2008, 2011). Alternatively, CRISPR-based strategies could be developed to destabilize donor centromeres (Zuo et al. 2017). Notably, donor genome removal is not strictly required, as panels of heterozygous diploid strains that carry homozygous loss-of-heterozygosity (LOH) tracts on a chromosome arm can be used for genetic mapping (Sadhu et al. 2016). Such LOH strains are equivalent to distal “CRI” recombinants prior to SPA (see Fig. 1, top). In diploid recombinants, local and distal repairs could be distinguished by sequencing, by screening for loss rather than gain of a marker, or by expression of a dosage-sensitive marker that differentiates one copy in local versus two copies in distal recombinants. Mapping results from CRI recombinants could differ from those from CRI-SPA isolates due to interactions with the diploid versus haploid background.

CRI-SPA-Map transfers alleles from a donor to a recipient strain. Therefore, allelic effects are only tested in the recipient background. As a consequence, allelic effects that depend on the donor background owing to epistasis may be missed.

We offer several recommendations for implementing CRI-SPA-Map. First, although CRI-SPA-Map rarely results in unexpected genome changes, we did observe some isolates that were aneuploid, diploid, or recombinant outside of the targeted area. To guard against phenotypic effects of these rare isolates, we recommend phenotyping multiple isolates from each targeted ORF.

We also advise engineering multiple ORFs surrounding specific variants or engineering densely and uniformly across the genome to detect ORFs with outlier phenotypes compared with neighboring ORFs. Introduction of donor variants is usually not limited to the targeted ORF, especially not in distal isolates, which carry all variants from the targeted ORF to the telomere. Therefore, the effects of a true causal variant are expected to be seen in local isolates from neighboring ORFs and in all distal isolates from ORFs between the causal variant and the centromere (Supplemental Fig. S8A–C).

Distinguishing local and distal isolates without sequencing requires a genetic marker at the telomere. Although this requires an engineering step, we do recommend separating the two types. Without selection for distal isolates, local isolates will likely predominate (only ∼12% of distal repairs were reported in the work of Sadhu et al. 2016). Identifying sufficient distal isolates would then require extensive sequencing. If local and distal isolates are not separated in ORF-based mapping, their expected association patterns will blend, rendering the data hard to interpret.

Although genotype-free ORF-based mapping was effective, sequencing a subset of the isolates further increased mapping resolution. Sequencing efforts could be directed at isolates at an association peak in genotype-free mapping.

The most frequent off-target effect of CRI-SPA-Map was rDNA recombination. Although these recombinants may simply be consequences of replication stress (D'Alfonso et al. 2024), we cannot rule out that CRI-SPA increases the frequency of rDNA recombination. Although excluding rDNA recombinants did not meaningfully alter our results for growth in liquid YPD, these recombinants may affect other phenotypes (Supplemental Fig. S12). We suggest sequencing a subset of isolates to detect recurrent recombination that may exist at other loci in a new strain pair. Such recombination can then be controlled by placing a selectable marker between the recombination locus and its respective telomere.

Bulk-segregant analysis of pooled CRI-SPA-Map isolates may be possible, but it would lose key advantages of our strategy here. Phenotyping with replication provided precise individual-level phenotypes. Combined with isolate genotypes, these enabled association mapping with linear models, yielding more interpretable effect sizes than the allele-frequency readouts of bulk-segregant analysis.

Finally, we recommend monitoring the fraction of distal compared with local colonies in the CRI-SPA-Map procedure. A disproportionately small fraction of distal isolates is a strong indicator that the targeted ORF lies on an unexpected chromosome.

CRI-SPA-Map has the potential to be expanded beyond its use here. The procedure is not limited to the YKO collection as the recipient. Any arrayed collection with selectable marker cassettes, with or without deletion of ORF sequences, could be used. Many arrayed insertion collections exist in a variety of species, which could allow the use of CRI-SPA-Map beyond *S. cerevisiae*. CRI-SPA-Map creates recombinants by introducing variants from a donor strain into a recipient strain without meiotic recombination and sporulation. Thus, it has the potential to create recombinants between infertile hybrid strains, perhaps even between species.

In conclusion, we have presented CRI-SPA-Map, a method for the efficient and effective introduction of donor DNA into a recipient genome. Panels of CRI-SPA-Map isolates enable high-resolution mapping of genetic effects on complex, quantitative phenotypes, even without genotyping. The method can be applied genome-wide, targeted to a single chromosome arm, or used for fine-mapping loci defined by other approaches. CRI-SPA-Map is a promising new tool for the dissection of the genetic basis of phenotypic variation.

## Methods

### Yeast strains, oligos, and media

Standard cloning, transformation, and strain engineering techniques are used throughout. All yeast strains and genotypes are listed in Supplemental Table S10, and the YKO strains used are listed in Supplemental Table S1. DNA oligo sequences used for plasmid and strain construction, genotyping, and sequencing are listed in Supplemental Table S11. Media formulations are in Supplemental Table S12. All yeast growth is at 30°C unless otherwise stated.

### CRI-SPA-Map strain engineering

Details of plasmid and strain engineering are described in the Supplemental Methods. We used BY4742 YKO strains with ORF deletions on the left arm of Chromosome XIV as the recipient strains (*MAT*α *his3*Δ*1 leu2*Δ*0 lys2*Δ*0 ura3*Δ*0* ynlxxxxΔ::KanMX) (Winzeler et al. 1999; Giaever et al. 2002). As the donor strain, we used a W303 strain (W8164-2C) (Reid et al. 2011) that we modified to contain mCherry::hphNT1 with the linker removed (Malcova et al. 2016) fused to the C terminus of YNL313C (at position ChrXIV:45308) to create YFA1746. HphNT1 carries a different terminator (*CYC1*) than the KanMX cassette used in the YKO collection (*TEF* from *Ashbya gossypii*), reducing opportunities for conversion of KanMX by HphNT1. We transformed strain YFA1746 with the CRISPR-Swap plasmid (pFA0055; Addgene 131774) to create YFA1743 (*MAT*a *can1-100 his3-11,15 leu2-3,112 trp1-1 ura3-1 CEN1–16::*p*GAL1-URA3*-*K. lactis* YNL313C*-*mCherry::hphNT1Δlinker).

### Production of the CRI-SPA-Map isolates

Available genome sequences of BY4742 (Cherry et al. 2012) and W303 (Matheson et al. 2017) were aligned to S288C and variants on ChrXIV-L identified using MUMmer (v 3.23) (Kurtz et al. 2004). We then identified 92 YKO ORFs including or neighboring these variants.

Cells of W303-SPA (YFA1743) and a BY4742 YKO strain were combined on a YPD plate and grown 8 h to mate. Approximately 30 µL of the mated pair was resuspended in 100 µL of sterile water, spread onto an SRC-Lys-Trp-Leu plate, and grown for 2 days to form diploid colonies that carry the pFA0055 plasmid. The raffinose stops the strong repression of the *GAL1* promoter.

Diploid colonies carrying the pFA0055 plasmid were gathered for a total volume of ∼30 µL and resuspended in 1 mL of sterile water. The cells were diluted 10,000-fold, and 100 µL was plated onto galactose-containing YPG plates to induce transcription from the *GAL1* promoter. After 3 days of growth, colonies formed, and the plates were replica-plated to SGC +5-FOA and grown for 2 days to ensure loss of *URA3* gene expression at each centromere.

To distinguish between local and distal repairs, each SGC+5-FOA plate was replica-plated to a YPD+Hyg plate and grown for 1 day. The percentage of distal repairs was determined by dividing the number of colonies growing on YPD+Hyg by the number of colonies growing on SGC+5-FOA (Supplemental Table S2). This percentage is likely an overestimate because the replicated colonies may contain both Hyg^S^ and Hyg^R^ cells. Colonies growing on SGC+5-FOA but not YPD+Hyg were saved as local repairs (Hyg^S^). Colonies growing on the YPD+Hyg plates were saved as distal repairs (Hyg^R^).

To isolate single repair events, at least eight Hyg^S^ and eight Hyg^R^ colonies were streaked for single colonies on YPD plates. Each single-colony purified isolate was tested for the following by growing on diagnostic media: (1) presence of the *LYS2* gene in the BY4742 background (SDC-Lys), (2) loss of the KanMX cassette (YPD+G418), (3) expected Hyg resistance (YPD+Hyg), (4) loss of the W303 chromosomes (SDC-Ura) and (5) loss of the CRISPR-Swap plasmid (SDC-Leu). All isolates used in further analyses were G418^S^, Lys^−^, Ura^−^, and Leu^−^. All local isolates were Hyg^S^, and all distal isolates were Hyg^R^.

To create wild-type isolates, BY4742 (Horizon Discovery YSC1049; YFA1608) was mated to the W303-SPA strain (YFA1743) and brought through the CRI-SPA procedure. As additional control strains for distal CRI-SPA-Map isolates, we engineered YFA1608 to have the HphNT1Δlinker cassette as described above. These additional control strains did not undergo the CRI-SPA-Map procedure.

### Genome sequencing and variant calling

We isolated genomic DNA from 400 µL YPD cultures grown overnight using the Quick-DNA 96 plus kit (Omega) after pretreatment of the cells with zymolyase (United States Biological). We prepared the genomic DNA for short-read sequencing on the Illumina platform using a modified Nextera (Illumina) based approach as previously described (Brion et al. 2020)

We created and pooled barcoded libraries of 555 isolates and several additional strains. The final pooled library had an average length of 585 bp and was sequenced with 150 bp paired-end reads using an Illumina NovaSeq 6000 instrument. Average quality scores were above 30. An average of 625,816 read pairs was obtained per sample, for a median read depth of 7.8 across the nuclear genome, with a range of 1.4 to 27 (Supplemental Table S2).

Sequencing reads were aligned to the S288C reference genome using BWA (v 0.7.17) (Li 2013). We used SAMtools (v 1.16.1) (Danecek et al. 2021) to remove reads tagged as duplicates, as unaligned, as mapping to more than one location, or with a mapping quality of less than 30. Haploid genotypes were called across all sequenced samples using BCFtools (v 1.16) to create a genome-wide VCF (Danecek et al. 2021). We retained variants with a quality score of at least 20.

### Identifying variants in the YKO strains

Using the genome-wide VCF, we retained variants that were called in a minimum of 90% of CRI-SPA-Map isolates with a quality score of 30 or more. We then identified variants with alternate allele calls in at least three isolates from the same YKO strain. We used Ensembl VEP (McLaren et al. 2016) release 115 to predict the consequences of the variants.

### Genotype screening of CRI-SPA-Map isolates

We designed criteria (for description, see Supplemental Methods) to detect unexpected outcomes of the CRI-SPA-Map procedure as well as potential strain contaminations or mislabeling. These criteria identify YKO strains with low percentage of HygR isolates, aneuploidy, mosaic tracts and tracts with heterozygous variant calls, BY variants in the targeted ORF, W303 variant tracts not spanning the targeted ORF, YKO strain impurity using DNA fingerprints, and recombination at locations other than ChrXIV-L. Isolates were removed from various analyses based on this screening.

### Locating tracts of W303 variants in the CRI-SPA-Map isolates

We identified runs of at least three consecutive variant calls with quality scores of 30 or more with the same parental (BY or W303) allele, discarded any variants with low-confidence allele calls (q < 30), and merged runs of the same allele. Runs on ChrXIV-L were extended to the telomere if they included at least one of the two most telomeric variants. The ChrXIV-L centromere was designated BY in all isolates.

### Measuring and analyzing growth rates in liquid YPD

Growth rates in liquid YPD were assayed using a Synergy H1 (BioTek Instruments) plate reader. The layout of the isolates on the plates and preculture growth conditions are described in the Supplemental Methods and Supplemental Table S2. Starter cultures of 400 µL YPD were grown overnight and then diluted in 100 µL of YPD to an approximate OD_600_ of 0.05 in a 96-well flat-bottom plate (Costar 3370). The plate was sealed with a Breathe-Easy membrane (Diversified Biotech) and grown for ∼18 h in the plate reader set at 29°C with OD_600_ absorbance readings every 6 min for 181 cycles with orbital shaking (1 mm) between reads. The growth of each local isolate was measured at least twice; the distal isolates, at least three times. The *MKT1-SAL1* locus strains were phenotyped in liquid YPD following the same procedure.

Growth rates from each well were determined using the Growthcurver R package (Sprouffske and Wagner 2016). Growth rates were defined as the number of doublings per hour at the inflection point of the growth curve and were corrected for potential plate effects (Supplemental Methods). Growth rates for isolates from one targeted ORF at a time were compared with the wild-type isolates. Multiple measurements were obtained per isolate. Data were analyzed using a mixed linear model:

Growthrate=genotype+isolate+error.

*Genotype* was modeled as a fixed effect indicating whether a measurement came from a wild-type or targeted-ORF isolate. *Isolate* was included as a random effect to account for systematic differences among isolates. The significance of *genotype* was assessed by ANOVA, with Bonferroni correction for the number of ORFs tested.

### Incorporation of genotype data in genetic mapping

For each DNA variant, all isolates carrying the W303 allele were compared with those carrying the BY allele. Bonferroni correction was applied for the number of analyzed DNA variants. To further assess significance, we performed 1000 random permutations, recorded the best *P*-value from each, and used the 95% percentile of these values as a permutation-based significance threshold.

Because of the fewer allele calls at the *sal1-1* variant compared with neighboring variants, we manually inspected read alignments to genotype this variant in 13 local and 12 distal isolates carrying nearby W303 variants. Because only this site was manually curated, it may have had slightly higher statistical power than other variants.

### Engineering and phenotyping of alleles at the *MKT1-END3-SAL1* locus

We created different allele combinations at the *MKT1-END3-SAL1* locus in BY4742 strain (YFA1608) with double-cut CRISPR-Swap (Lutz et al. 2019). The repair fragments were created by splicing overlap extension PCR (Horton et al. 1989). Details are described in the Supplemental Methods.

The engineered *MKT1-END3-SAL1* locus strains were randomized, pinned, and phenotyped at 384-colony density on solid agar plates using a ROTOR+ and PIXL (Singer Instruments). Plate pouring, strain randomization, growth, and phenotyping are described in the Supplemental Methods.

Colony sizes were corrected for plate identity and position on the plate (Supplemental Methods). The standard environmental condition (solid YPD) was compared to one other environment at a time. We analyzed the effects of the causal variants in *MKT1* and *SAL1* using the following linear model:

y=MKT1_genotype∗SAL1_genotype∗Environment+Strain+error.

Here, *y* denotes the phenotype (liquid growth rates or colony sizes); *MKT1_genotype* and *SAL1_genotype* are fixed effects denoting whether a given strain carried the BY or W303 allele at the *MKT1*-D30G or *SAL1* variant; *Environment* denotes a fixed effect for the environment (either the standard condition or the other condition); and *Strain* is a random effect denoting strain identity. The model included all interaction terms between the fixed effects, allowing simultaneous determination of the marginal effects of each individual allele, G×G between alleles, G×E for each allele, and the global G×G×E term that asks if the pattern of epistasis (G×G) depends on the environment. The statistical significance of the G×G×E term and of the G×E terms for each allele was gauged using ANOVA.

The statistical significance of the G×G term was assessed using models of one condition at a time:

y=MKT1_genotype∗SAL1_genotype+Strain+error.

Marginal effects of each allele and of multiallele constructs compared with the wild-type BY strain were tested as described under “ORF-based genetic mapping.” No multiple-test correction was performed for these analyses. Instead, raw *P*-values were reported and interpreted, keeping in mind the exploratory nature of these G×E experiments.

### Code availability

Unless otherwise stated, all analyses were performed in R (R Core Team 2025). Analysis code is available at GitHub (https://github.com/lawls18/CRI-SPA-Map) and as Supplemental Code.

## Data access

Illumina sequence data generated in this study have been submitted to the NCBI BioProject database (https://www.ncbi.nlm.nih.gov/bioproject/) under accession number PRJNA1374440.

## Supplemental Material

Supplement 1

Supplement 2

Supplement 3

Supplement 4

Supplement 5

Supplement 6

Supplement 7

Supplement 8

Supplement 9

Supplement 10

Supplement 11

Supplement 12

Supplement 13

Supplement 14

Supplement 15
