# Supplementary material for: High-resolution genotype-free mapping of genetic variation with CRI-SPA-Map
Source: Genome Res. 2026 May;36(5):1016–28. doi: 10.1101/gr.281514.125 (PMC13445715; doi:10.1101/gr.281514.125)
Supplement: Supplementary file 1 [file Supplemental_Material.pdf]

Table of Contents

Supplemental Methods: pages 2–12

Supplemental References: pages 13–14

Supplemental Figures S1–S12: pages 15–26

Supplemental Methods

Plasmid and strain engineering

Construction of mCherry::HphNT1Δlinker plasmid: We removed the linker sequence within the mCherry-HphNT1 cassette from pFM689 (BFA0135, Addgene #74635 (Malcova et al. 2016)) by restriction digestion with BamHI and BglII to release the intervening sequence and then ligated the compatible DNA overhangs to form the plasmid mCherry::HphNT1Δlinker (BFA0262).

Engineering the W303 donor strain: We PCR amplified the mCherry::HphNT1Δlinker cassette with primers OFA1369 and OFA1370 and then used this DNA fragment to transform the W303 SPA strain. The location of the mCherry::HphNT1Δlinker insertion at the 3'-end of *EMW1* and the absence of an *EMW1* allele without the insertion was verified by PCR.

Engineering the *MKT1-END3-SAL1* locus: We created different combinations of *MKT1* and *SAL1* alleles in the BY4742 background by engineering the region on ChrXIV-L that includes *MKT1*, *END3* and *SAL1* with double-cut CRISPR-Swap (Lutz et al. 2019). First, we replaced *MKT1* with a KanMX cassette to create YFA1822 by amplifying the KanMX fragment from YFA0007 with OFA1283 and OFA1284 and transforming it into BY4742 (YFA1608). The presence of KanMX replacing the *MKT1* ORF was verified by colony PCR with OFA1365 and OFA0036 and the absence of the *MKT1* allele with OFA1365 and OFA1366. Next, we replaced *SAL1* with a HphMX cassette by amplifying the HphMX cassette from YLK1950/YFA0006 with OFA1471 and OFA1472 and transforming it into BY4742 mkt1Δ::kanMX (YFA1822). The presence of HphMX replacing *SAL1* was verified by colony PCR with OFA1473 and OFA0036 and the absence of *SAL1* with OFA1473 and OFA1474. The BY4742 mkt1Δ::kanMX sal1Δ::hphMX (YFA1831) strain was then transformed with ~100 ng of the CRISPR-Swap plasmid (pFA0055) and ~1 µg of PCR generated alleles of the 6,275-bp *MKT1*, *END3* and *SAL1* locus.

The allele fragments were created using splicing overlap extension PCR (Horton et al. 1989) as follows. The BY and W303 alleles were amplified from YFA1608 and YFA1718 genomic DNA, respectively using primers OFA1365 and OFA1475. *MKT1*-30G was amplified from W303 DNA with OFA1497 and OFA1366 and BY gDNA with OFA1498 and OFA1501. SAL1-nfs was amplified from BY DNA with OFA1497 and OFA1499 and from W303 DNA with OFA1500 and OFA1501. *MKT1*-30G+SAL1-nfs was amplified from W303 DNA with OFA1497 and OFA1366; and OFA1500 and OFA1501 and from BY DNA with OFA1498 and OFA1499. An additional BY allele was created as described for the *MKT1*-30G+SAL1-nfs allele, except the template was BY for each of the three reactions.

Amplicons were run on a 1% agarose gel and the fragments were purified by gel extraction (NEB). To create the final alleles, the fragments were combined by amplifying with OFA1365 and OFA1475. All reactions were run on a 1% agarose gel and purified prior to transformation.

Whole-genome sequencing

We sequenced 555 isolates as well as several additional strain types: 1) six wild-type isolates that carry the BY nuclear genome, 2) three BY4742 strains with *EMW1* tagged with *mCherry::hphNT1∆linker* used as controls for distal CRI-SPA-Map isolates, and 3) four W303 SPA strains containing *mCherry::hphNT1∆linker* or HphMX tags at various genome locations. These additional strains were used for calling high-quality variants as described below.

The isolates and strains were arrayed in six 96-deep-well plates (Supplemental Table S2) in 500 µl YPD and grown overnight at 30 ºC on an Eppendorf MixMate shaker at 1100 rpm. After growth, each plate was centrifuged at 3700 rpm for 5 min to collect the cells. To remove the supernatant, plates were inverted over a collection container and while still inverted, placed onto paper towels to allow excess liquid to be removed. The cells were resuspended in 800 µl of a solution containing 1 M sorbitol, 0.1 M EDTA, 14.3 mM β-mercaptoethanol, and 4 units of Zymolyase lytic enzyme (United States Biological) and sealed with a foil seal and incubated for 2 hrs at 37 ºC with occasional mixing to digest the cell walls. The cells were then centrifuged and the supernatant discarded as described above.

We isolated genomic DNA using the Omega, Quick DNA 96 Plus Kit following the Biological fluids and Cells protocol. After completing the DNA extraction protocol, we added 30 µL of DNA elution buffer (10 mM Tris-HCl, pH 8.5, 0.1 mM EDTA) prewarmed to 55 ºC to each well and incubated the plates at room temperature for 3 min prior to centrifugation. The concentration of the eluted DNA in each well was determined using the Qubit dsDNA BR assay kit (Thermo Fisher Scientific) in a 96 well format using a Synergy H1 plate reader (BioTek Instruments) using a self-made standard curve.

We prepared the genomic DNA for short-read sequencing on the Illumina platform using a modified Nextera (Illumina) based approach as previously described (Brion et al. 2020). Each 96-well plate of genomic DNA was processed alone or with one other plate. We diluted the genomic DNA to 5 ng in 11 µl in the wells of a new PCR plate and then added 4 µl of Tagment DNA buffer and 5 µl of Tagment DNA enzyme (diluted 1:20 in tagment DNA buffer). The tagmentation reaction plates were covered with PCR film and incubated at 55 ºC for 10 minutes in a SimpliAmp thermal cycler (Thermo Fisher Scientific). Each sample of tagmented genomic DNA was then amplified with unique Illumina i5 and i7 index primer pairs to allow pooling of all samples prior to sequencing (Supplemental Table S2). The amplification reactions contained 10 µL of the tagmentation reaction, 2 µL of a 5-µM index primer pair mixture, 5 µL of ExTaq buffer, 4 µL of dNTPs, 0.375 µL of ExTaq polymerase (Takara), and 29.625 µL of sterile molecular biology grade water. We then sealed the plates and ran the amplification reactions in a SimpliAmp thermal cycler (Thermo Fisher Scientific) using the following parameters: initial denaturation at 95 ºC for 30 seconds, then 17 cycles of 95 ºC for 10 seconds, 62 ºC for 30 seconds, and 72 ºC for 3 minutes.

We pooled 10 µl of each amplified sample from one plate and ran 200 µl of this mixture on a 2% agarose gel and excised the DNA in the 450–550 bp size range. We then purified the DNA from the gel slice using the Monarch Gel Extraction Kit (NEB). We determined the concentration of

DNA in the pooled libraries from each of the six plates using Qubit and then diluted each pool to 10 ng/µl. We then combined 5 µl from each of the six pools to create the final library pool.

The average fragment size of the final pooled library was 585 bp, as determined using an Agilent TapeStation. High-output sequencing of 150-bp paired-end reads was performed on an Illumina NovaSeq 6000 instrument SP run at the University of Minnesota Genomics Core. The average quality scores were above 30 and an average of 625,816 read-pairs were obtained for each sample. The median read depth across the nuclear genomes was 7.8, with a range of 1.4 to 27 across all samples (Supplemental Table S2).

Identifying variants between the BY4742 and W303 SPA strains

Analyses in this section used GNU parallel (Tange 2022). We used available genome sequences of BY4742 (http://sgd-archive.yeastgenome.org/sequence/strains/BY4742/BY4742_Toronto_2012 at SGD, (Cherry et al. 2012)) and W303 (Matheson et al. 2017) to determine the nucleotide variants on chrXIV-L. We aligned the chrXIV-L sequences from BY4742 and W303 to the S288C reference genome (sacCer3 R64-5-1) and used show-snps to create two variant lists using MUMmer (v 3.23) (Kurtz et al. 2004). These lists were then transformed into VCFs using a custom Python script. The VCFs were then combined to identify the BY4742/W303 chrXIV-L variants. We identified 445 variants and two breaks in synteny due to Ty polymorphisms (Supplemental Table S1). We used these variant positions to select the YKO strains with the nearest KanMX cassette for CRI-SPA-Map.

To identify any variants present between BY4742 (the background of the strains in the deletion collection) and the S288C reference genome, we used a custom R (v 4.4.3) script to extract the columns of the VCF of the six wild-type isolates and the three BY4742 strains containing *EMW1*-mCherry::hphNT1Δlinker. After filtering for variant sites at which 75% or more of these nine BY4742 isolates and strains carried the same alternative allele, we identified 86 sequence differences between BY4742 and S288C. We then used the same criteria on the four sequenced W303 SPA strains and found 8,305 sequence differences between them and the S288C reference. Comparison of these two variant lists identified 67 variants that were shared between BY4742 and W303 SPA but different from the S288C reference. This left 8,257 variants across the nuclear genome to distinguish BY4742 from W303 SPA with 377 variants on chrXIV-L (Supplemental Table S3).

To determine mitochondrial variants between BY4742 and S288C, we analyzed the mitochondrial sequences of the three BY4742 strains containing *EMW1*-mCherry::hphNT1Δlinker, which all carry the same BY4742 mitochondrial genome. We did not analyze the six wild-type isolates due to the mitochondrial shuffling that occurs during the mating step of CRI-SPA-Map. After filtering for variant sites at which all three BY4742 strains had the same alternative allele, there were five mitochondrial variants between BY4742 and S288C. We then compared the four sequenced W303 SPA strains to S288C and found 53 mitochondrial variants called in at least 75% of the W303 SPA strains. All five mitochondrial

variants identified between BY4742 and S288C were also present between W303 SPA and S288C, leaving us with 48 variants to distinguish the BY4742 and W303 SPA mitochondrial genomes (Supplemental Table S3).

Identifying variants in the YKO strains

Using the genome-wide VCF described above, we retained variants that were called and had a genotype quality score of at least 30 in a minimum of 90% of the 555 sequenced CRI-SPA-Map isolates. To investigate whether any of these variants likely arose before CRI-SPA-Map and were therefore present in the original YKO strain, we looked for any remaining variants that had at least three isolates from the same targeted ORF with alternate allele calls. After identifying these variants, we manually removed the variants that arose from YKO strains with a low percentage of Hyg^R colonies or from the Lys^+ YNL268W YKO strain.

This criteria identified 69 variants that are different between S288C and BY, 9 fingerprint variants (described below), and 86 strain-specific background variants (Supplemental Table S9). We used Ensembl VEP (McLaren et al. 2016) release 115 to predict the consequences of the variants.

Criteria for screening CRI-SPA-Map isolates

Strains with low percentage of HygR isolates: After replica-plating colonies to YPD + Hyg, an average of 33% of colonies were Hyg^R (Supplemental Table S2). Seven YKO strains had notably lower percentages (0 – 7%). A likely reason for this low recovery of Hyg^R isolates is that these YKO strains carried deletions of genes different from those we targeted. Specifically, if the KanMX cassette was on a different chromosome arm than ChrXIV-L, the HphNT1Δlinke*r* cassette on ChrXIV-L would not be introduced into the given isolate during repair of the double-strand break. In this scenario, Hyg^R colonies would be rare, as they can only be obtained from infrequent spontaneous breaks and repairs on ChrXIV-L. All sequenced isolates from the seven YKO strains with few Hyg^R colonies were also flagged by sequencing-based analyses (see below), further supporting that these isolates arose from YKO strains with gene deletions on other chromosomes. All 112 isolates arising from the seven YKO strains with low Hyg^R recovery were flagged according to this criterion.

Aneuploidy: To determine if the CRI-SPA-Map isolates exhibited large-scale copy number variants (CNVs), we used CNVnator (v 0.4.1) (Abyzov et al. 2011) to divide the chromosomes of the S288C reference genome into non-overlapping 5,000 bp bins and to identify bins with read depth differing from the average read depth across the nuclear genome (Supplemental Table S2). Isolates were considered aneuploid when they contained regions of higher or lower read depth (“eval-1” value reported by CNVnator < 0.00000539, corresponding to 0.05 / the total number of CNVs reported by the software) that extended for at least half of the given chromosome. We identified 11 sequenced isolates with aneuploidy (Supplemental Fig. S4).

Of the six sequenced CRI-SPA-Map isolates from the YNL079C (*TPM1*) YKO strain, five exhibited a duplication on chromosome IX. Upon closer examination, the 6th isolate also showed a duplicated segment just below half of the length of chromosome IX. Thus, all sequenced isolates from the YNL079C YKO strain showed evidence for duplication of this chromosome, which contains *TPM2*, the paralog of the deleted *TPM1* gene. Therefore, we flagged all 24 isolates derived from YNL079C. An additional five isolates from three additional YKO strains showed evidence for aneuploidy. In total, we removed 29 isolates from four YKO strains.

Mosaic tracts and tracts with heterozygous variant calls: We flagged 26 isolates from 22 ORFs because they showed more than one W303 variant track on ChrXIV-L. Analysis of these variant tracts revealed two categories: mosaic tracts and heterozygous variant call tracts.

Mosaic tracts were observed in 18 isolates (Supplemental Fig. S5). We defined these mosaic tracts as tracts of homozygous W303 variants interrupted by tracts of at least three homozygous BY variants. The isolate YNL099C_S2 has a tract of W303 variants adjacent to but not spanning the YNL099C ORF and would normally be flagged for not having W303 variants spanning the ORF. However, there is one BY variant between this W303 tract and the ORF and there are no additional variants for 22,302 bp. All other isolates of this YKO strain have tracts of W303 variants spanning the ORF and all isolates had matching DNA fingerprints (see below). We conclude that this isolate has a mosaic tract arising from a correctly targeted ORF.

Heterozygous variant calls were observed within the repair tracts of 8 isolates. These isolates were homozygous for W303 alleles in the targeted ORF and had runs of at least six variants with heterozygous calls showing both BY and W303 alleles. The rest of their genomes were homozygous for BY alleles. We attempted to mate 4 of these isolates to both MATa and MATα strains and observed mating to only MATa. All of the isolates flagged by this analysis had distal repairs, likely due to the difficulty of confidently calling a short heterozygous tract that may have arisen from local repair. We noticed that the isolates with heterozygous tracts grew faster than isolates from the same YKO strain without apparent heterozygous tracts. We hypothesize that these isolates are MATa diploids that underwent separate DNA repairs on two BY chromosomes present in a tetraploid or during the G2 phase of the cell cycle, followed by an error in chromosome segregation. After SPA, the cell then became diploid.

BY variants in the targeted ORF: Because the YKO strains carry KanMX cassettes instead of the sequence of the deleted gene, CRI-SPA-Map isolates derived from YKO strains should have W303 alleles at the targeted gene. Therefore, isolates with a BY allele call at a variant within the targeted ORF were flagged in this analysis, as such calls suggest that the targeted ORF had not been deleted in the BY YKO strain used in the CRI-SPA-Map cross. Instead, the KanMX cassette had likely been present (and was then replaced by CRI-SPA-Map), at a different gene than the targeted one. For YKO strains with ≥ 75% of sequenced CRI-SPA-Map isolates with BY variants in the targeted ORF, we flagged all Hyg^S and Hyg^R isolates, sequenced or unsequenced, as we assumed that the majority of these isolates were not from the intended deletion strain. All 72 isolates from four YKO strains were flagged based on this criterion, of a total 80 isolates from 10 YKO strains.

W303 variant tracts not spanning the targeted ORF: For the targeted ORF YNL133C, none of the observed tracts of introduced W303 variants in local isolates spanned this ORF. These isolates are likely from the YNL092W YKO strain, as their DNA fingerprints (see below) and repair tract positions matched isolates from YNL092W. The YNL133C and YNL092W strains are in adjacent wells in the YKO collection, suggesting possible cross-contamination. We flagged all 16 isolates (Hyg^S, Hyg^R, sequenced, and unsequenced) from YNL133C.

YKO strain impurity using DNA fingerprints: We identified nine sequence variants that segregated among the CRI-SPA-Map isolates (Supplemental Table S9). For eight of these variants, the alternative allele was observed in half (range of 46 – 53%) of our isolates, and the remaining variant was observed in 16% of our isolates.

One method by which strains were created in the YKO collection was to replace the ORF with the KanMX cassette in a BY4741 / BY4742 diploid followed by sporulation. This method was used to create 70% of the haploid YKO strains (Giaever et al. 2002). Thus, any DNA variants between BY4741 and BY4742 would be expected to segregate in the YKO collection. To test this, we performed BLASTN (BLAST+ tools (Camacho et al. 2009)) searches using each of the nine variants and flanking sequences to query databases created from partial genome sequences of BY4741 and BY4742 (Song et al. 2015). We found that the BY4741 and BY4742 differ at the eight variant positions with the highest alternative allele frequency (positions in S288C are chrI:41475 G/T, chrIV:769328 G/A, chrVIII:499958 C/T, chrVIII:511255 C/T, chrXIV:189585 C/T, chrXV:773264 G/C, chrXVI:667520 T/G, and chrXVI:677102 A/G). The origin of the lower frequency variant (chrXI:589175 T/A) is unknown.

We used these nine variants as a DNA fingerprint to verify that isolates obtained from the same deletion strain have the same variant call at each fingerprint position. A consensus DNA fingerprint was determined for each deletion strain when at least five of the six sequenced isolates had the same variant calls. If a consensus DNA fingerprint could not be determined, all isolates from this YKO strain were flagged. If only one isolate did not match the consensus fingerprint at more than one of the nine positions then this isolate was flagged. This analysis flagged 131 isolates from 11 ORFs.

Recombination at locations other than ChrXIV-L: We searched for mitotic recombination events on chromosome arms other than ChrXIV-L by locating tracts of W303 variants elsewhere in the genome. This analysis suggested mitotic recombination at locations other than ChrXIV-L in 42 CRI-SPA-Map isolates from 28 YKO strains. Three isolates had W303 variant tracts on two chromosome arms other than ChrXIV-L (Supplemental Fig. S3).

Nearly half of these 42 isolates had W303 variant tracts on the right arm of chromosome XII (chrXII-R; 9 Hyg^S and 10 Hyg^R isolates). In these 19 isolates, the tracts of W303 variants extended from near the rDNA locus to the telomere of chromosome XII-R, suggesting increased mitotic recombination at this highly repetitive locus.

Due to the length and frequency of these recombination events on chrXII, we wanted to exclude all isolates that carry these events, including those that were not sequenced. This was possible due to the existence of a polymorphism in the *HAP1* gene located between the rDNA array and

the chrXII-R telomere. BY strains have a Ty1 insertion at the 3’-end of the *HAP1* ORF (*hap1-Ty1*), while the W303 *HAP1* gene is wild-type (Gaisne et al. 1999). BY strains with the *hap1-Ty1* allele are more susceptible to fluconazole than strains with the *HAP1* allele (Saha et al. 2024).

To screen for mitotic recombination of chrXII-R, all isolates (sequenced and non-sequenced) were pinned from glycerol stocks to YPD PlusPlates using a ROTOR+ instrument (Singer Instruments, Roadwater, United Kingdom). After three days of growth, the colonies were pinned with the ROTOR to Plus plates (Singer Instruments) containing SDC + 16 µg/ml fluconazole (Cayman Chemical) and incubated at 30˚C for two days. Colony size was then determined using a PIXL® instrument (Singer Instruments). Local isolates were considered likely to carry the wild-type, W303 *HAP1*, allele if their colony size was at least 30% larger than the median colony size of all local isolates from the corresponding YKO strain. For distals, the colony needed to be at least 20% larger to be considered. The genotypes of such isolates were then verified by PCR using primers specific to each *HAP1* allele (primers OFA1691 and OFA1692 amplified wild-type *HAP1* and OFA1691 and OFA1693 amplified *hap1-Ty1*. This screen identified 24 additional *HAP1* strains (5 Hyg^S and 19 Hyg^R).

Across all 1,451 screened isolates, we identified 40 (2.8%) that had mitotic recombination on chrXII that likely extends from the rDNA locus to the telomere (Supplemental Table S4). We did not detect mitotic recombination in 16 wild-type isolates that went through CRI-SPA-Map without a Cas9/gRNA cleavage. It is possible that CRI-SPA-Map increases the frequency of mitotic recombination.

Other filtering: Two isolates (YNL291C_S2 and YNL301C_R2) were flagged for having unexpected tracts of W303 variants based on their hygromycin susceptibility. YNL291C_S2 had a distal tract and YNL301C_R2 had a local tract. This suggests a possible strain mix-up after testing growth on YPD + Hyg and before sequencing.

Filtering summary: Application of these analyses to the 1,451 isolates without auxotrophy issues resulted in flagging of 273 isolates across 65 YKO strains. Of these, 139 isolates (51%) from 37 YKO strains were flagged by analyses that did not require sequencing. A large majority of the flagged isolates (209 from 18 YKO strains, 77%) were flagged due to likely problems with our copy of the YKO collection. There were 1,178 (81%) isolates spanning 78 YKO ORFs without any flags Supplemental Table S2.

Locating tracts of W303 variants in the CRI-SPA-Map isolates

DNA variant calls were simplified based on the called genotype and genotype quality score as follows: a site with a quality score of zero was designated as “no call”, a call with genotype quality score higher than zero but below 30 was designated as “low quality,” and a call with a score of at least 30 was designated as either “BY” or “W303” depending on the called allele. An additional variant was added marking the centromere of ChrXIV and each isolate was assigned “BY” at this site. Using the resulting allele designations at the 377 variants on ChrXIV-L plus the ChrXIV centromere call, we identified runs of at least three consecutive variant calls with the

same parental (BY or W303) allele. We discarded “no call” and “low quality” calls and subsequently merged any consecutive runs of the same parental allele. For each isolate, we determined the genomic coordinates of the start and end of each run using the variant locations marking the start and end of the identified tracts. If a run began at either of the two most telomeric variants on ChrXIV-L, we considered the run to start at the end of the chromosome.

To estimate the length of the W303 DNA segment introduced into the BY background, we used variant runs on ChrXIV-L to determine the distance between the targeted ORF and the 5’ and 3’ ends of the repair tracts. For example, moving 5’ from the start codon of the ORF we identified the farthest W303 variant call in a run of W303 variants. The distance between the start of the ORF and this farthest W303 variant position is the minimal repair length on the 5’ end of the ORF. The maximal repair tract length is the distance from the start of the ORF to the first BY variant in a run of BY variants. We calculated at least one repair tract length in 286 isolates. If the run of W303 variants did not extend outside of the ORF, the isolate was not considered. This results in a conservative overestimate of the median minimal amount of introduced W303 DNA, because very short W303 DNA tracts that replace the ORF but no flanking variants are not considered. Additionally, we removed all isolates derived from the 11 YKO strains in which the deleted ORF did not fully lie between our most telomeric and centromeric BY/W303 variants, as the lack of variants near these 11 ORFs would likely cause an overestimate of repair tract lengths. Finally, we excluded any isolates flagged for unexpected auxotrophies or issues suggestive of deletion collection problems (low Hyg^R, BY ORF variants, W303 variants not spanning ORF, mosaic tract, heterozygous reads, and inconsistent deletion strain fingerprints).

Quantification of meiotic linkage block lengths

We analyzed genotype data from 1,012 meiotic segregants from the BY/RM cross created in (Bloom et al. 2013). These segregants had previously been whole-genome sequenced to low coverage, and genotypes had been imputed at each of 42,052 variants. We extracted 74,726 runs of BY or RM genotype calls from the data and computed the distance between the markers delimiting these runs.

Measuring growth rates in liquid YPD

Growth rates in liquid YPD were assayed using a Synergy H1 (BioTek Instruments) plate reader. Each of the Hyg^S isolates were arrayed at least once on one of nine 96-well plates. Each of the nine plates contained the same 16 CRI-SPA-Map wild-type strains and 3 blank wells arrayed in different positions. The Hyg^R isolates were arrayed once on one of eight 96-well plates. On each Hyg^R plate were the same 6 CRI-SPA-Map wild-type strains and the same 4 BY4742 *EMW1-mCherry::hphNT1∆linker* wild-type strains and 2–3 blank wells arrayed in different positions. The nine Hyg^S plates were phenotyped twice and the eight Hyg^R plates were phenotyped three times.

Prior to phenotyping, all isolates were pinned from glycerol stocks to YPD or YPD + Hyg agar for the local and distal isolates, respectively. After 3 days of growth at 30 ºC the plates were stored at 4 ºC for up to two weeks.

We inoculated each culture for the plate reader runs with actively growing starter cultures. For the first run of a plate, starter cultures of 400 µl of YPD in a 2-ml deep-96-well-plate were inoculated with cells from the agar plates stored at 4 ºC. These starter cultures were then sealed with foil and stored at 4 ºC until they were used to inoculate new starter cultures for the second and third plate reader runs. To grow the starter culture plate, we sealed it with a Breathe Easy membrane (Diversified Biotech) and placed it on an Eppendorf MixMate set at 1100 rpm. After overnight growth at 30 ºC, we diluted the starter cultures in 100 µl of YPD to an approximate OD_{600} of 0.05 in a shallow 96-well plate. We then sealed the plate with a Breathe Easy membrane and placed it in the plate reader.

The strains were grown for ~18 h in the plate reader set at 29 ºC with OD 600 absorbance readings taken every 6 min for 181 cycles with orbital shaking (1 mm) between reads.

Growth rates were determined using the Growthcurver R package (Sprouffske and Wagner 2016) to fit growth curves to each well and identify the inflection point at which the yeast culture begins to exit exponential growth. The growth rates were then defined as the number of doublings per hour at the inflection point. Statistical analyses used the $\log_2$ of the growth rate (“growth rate” hereafter).

To avoid possible confounding by systematic differences between plates, we corrected growth rate for the specific plate they were grown in using the following mixed linear model:

growth rate = genotype + Isolate + plateName + error

Growth rate was used as the dependent variable, modeled as the sum of a series of random effects including the plate the measurement stemmed from. To fully represent the data during pre-processing, the model also included random effect terms for the genotype (i.e. the targeted ORF) and the isolate from which a given measurement was taken. We obtained corrected phenotypes by subtracting the effects of *plateName* from the raw measurements and used these residuals in further analyses.

Phenotyping on solid agar plates

Strains from the *MKT1-END3-SAL1* locus engineering were pinned, randomized and colony sizes measured using a ROTOR+ pinning robot and a PIXL instrument (Singer Instruments). Details regarding instrument settings and consistent PlusPlate (Singer Instruments) preparation are described below.

Glycerol stocks of the engineered *MKT1-END3-SAL1* strains were thawed and pinned from glycerol stock plates to YPD agar PlusPlates using the ROTOR+ to create the source plates. The strains on each plate were then randomized using the PIXL Re-Array workflow onto new YPD PlusPlates to reduce the impact of edge and plate effects. Two unique random arrays were

created from each source plate. The randomization uses Well-Plate Maker (WPM) (Borges et al. 2021) and additional corrections to avoid the following: colonies of the same strain being neighbors, wild-type strains being unevenly distributed across different plates, a given strain being on an edge position in both random arrays, and any one strain being on edge positions more frequently than any other strain. These random array plates were then used to inoculate cultures for glycerol stocks.

The glycerol-stock plates containing the randomized arrays were thawed and pinned using the ROTOR+ to 96 density YPD agar PlusPlates to create “pre-activation plates”, which were incubated at 30 ºC for 18 – 36 hours. Pre-activation plates were stored at 4 ºC for up to 3 days before use. Pre-activation plates were pinned using the ROTOR+ to 384 density PlusPlates containing YPD medium to create “activation plates”, which were incubated at 30 ºC for 20 – 24 hours. Immediately after incubation, activation plates were pinned using the ROTOR+ to 384 density PlusPlates containing the conditional medium to create “phenotyping plates”, which were incubated at 30 ºC for exactly 40 hours. Immediately after incubation, the phenotyping plates were analyzed using the PIXL.

PlusPlate preparation: We used the following procedure for the preparation of PlusPlates for use in the ROTOR+ and PIXL to ensure the plates were level and uniform. To prepare the media, yeast extract (1%) and peptone (2%) were dissolved in deionized water. This YP medium was divided into bottles with stir bars and autoclaved for 30 minutes. Immediately after autoclaving, 20% glucose solution (2%) was added to each bottle along with non-heat sensitive additives and the bottles were shaken to mix. Heat sensitive additives were added after YPD cooled to 60 ºC and mixed by stirring. Media was allowed to cool in a 53 ºC incubator for approximately 2 hours before plates were filled.

To fill the PlusPlates, a 1 L bottle with 500 mL of 53 ºC molten agar media was put on a stir/hot plate set to low heat and was stirred without producing bubbles. PlusPlates were laid out on a level surface. A 50-mL serological pipette was used to draw 55 mL of media, and exactly 50 mL were dispensed into each plate. The remaining 5 mL were dispensed back into the bottle. These steps were repeated until all media was used. The filled plates were allowed to cool and dry for 18 to 24 hours before either being used or they were inverted and bagged for longer term storage. Plates were not used after being stored for more than three weeks.

Yeast pinning: Plates were pinned using the ROTOR+. Plates were pinned from 96 density liquid arrays to 96 density solid arrays with 96 density long RePads (Singer Instruments) using default settings except the following: 10% target pin pressure, 2.25 mm target overshoot, 1 wet mix rotation cycle. Plates were pinned from 96 density solid arrays to 384 density solid arrays with 96 density short RePads using default settings except the following: no recycle, automatic offset, 15% target pin pressure, 2.25 mm target overshoot. Plates were pinned from 384 density solid arrays to 384 density solid arrays with 384 density short RePads using default settings except the following: automatic offset, 2.25 mm target overshoot.

Measuring colony size: The PIXL’s Colony Detection protocol was used to photograph the plates and perform image analysis to identify yeast colonies and measure colony sizes. A

Colony Detection template was used with the following settings: Background Subtraction algorithm, *S. cerevisiae* species, and 0.5-3.0 mm radius filter. The PIXL's blackout screen was inserted prior to image acquisition. If colonies were too small to be detected using the Background Subtraction algorithm but larger than 0.5 mm, the process was repeated using the Colony Separation algorithm. Raw image analysis data from the PIXL was processed so that each colony was matched to its strain information. The output from these procedures was the area of each colony, measured in mm^2.

Correction for edge and plate effects: Colonies at the edge of solid agar plates grew larger than those in the center of the plate, a well-known phenomenon in solid plate-based phenotypic assays (Wagih et al. 2013). Our randomization strategy (see above) was designed to avoid confounding of genotype with plate position. In addition, we corrected colony sizes for colony position on the plate using the following mixed linear model:

colSize = genotype + Strain + plateName + Row + Column + error

In this model, we used colony size as the dependent variable, modeled as the sum of a series of random effects including the row and column in which a colony was located, the plate a colony was on (to control for systematic differences between plates). Rows and columns were modeled as categorical factors. To fully represent the data during pre-processing, the model also included random effect terms for the genotype (i.e. the introduced DNA fragment in the validation experiments) and strain of a given colony. We obtained corrected colony sizes by subtracting the effects of *plateName*, *Row*, and *Column* from the raw measurements and used these residuals in further analyses.

Supplemental References

Abyzov A, Urban AE, Snyder M, Gerstein M. 2011. CNVnator: An approach to discover, genotype, and characterize typical and atypical CNVs from family and population genome sequencing. *Genome Res* **21**: 974–984.

Bloom JS, Ehrenreich IM, Loo WT, Lite T-LV, Kruglyak L. 2013. Finding the sources of missing heritability in a yeast cross. *Nature* **494**: 234–237.

Borges H, Hesse A-M, Kraut A, Couté Y, Brun V, Burger T. 2021. Well Plate Maker: a user-friendly randomized block design application to limit batch effects in large-scale biomedical studies ed. J. Kelso. *Bioinformatics* **37**: 2770–2771.

Brion C, Lutz SM, Albert FW. 2020. Simultaneous quantification of mRNA and protein in single cells reveals post-transcriptional effects of genetic variation. *eLife* **9**: e60645.

Camacho C, Coulouris G, Avagyan V, Ma N, Papadopoulos J, Bealer K, Madden TL. 2009. BLAST+: architecture and applications. *BMC Bioinformatics* **10**: 421.

Cherry JM, Hong EL, Amundsen C, Balakrishnan R, Binkley G, Chan ET, Christie KR, Costanzo MC, Dwight SS, Engel SR, et al. 2012. Saccharomyces Genome Database: the genomics resource of budding yeast. *Nucleic Acids Research* **40**: D700–D705.

Gaisne M, Bécam AM, Verdiere J, Herbert CJ. 1999. A “natural” mutation in Saccharomyces cerevisiae strains derived from S288c affects the complex regulatory gene HAP1 (CYP1). *Current Genetics* **36**: 195–200.

Giaever G, Chu AM, Ni L, Connelly C, Riles L, Véronneau S, Dow S, Lucau-Danila A, Anderson K, André B, et al. 2002. Functional profiling of the Saccharomyces cerevisiae genome. *Nature* **418**: 387–391.

Horton RM, Hunt HD, Ho SN, Pullen JK, Pease LR. 1989. Engineering hybrid genes without the use of restriction enzymes: gene splicing by overlap extension. *Gene* **77**: 61–68.

Kurtz S, Phillippy A, Delcher AL, Smoot M, Shumway M, Antonescu C, Salzberg SL. 2004. Versatile and open software for comparing large genomes. *Genome Biol* **5**: R12.

Lutz S, Brion C, Kliebhan M, Albert FW. 2019. DNA variants affecting the expression of numerous genes in trans have diverse mechanisms of action and evolutionary histories ed. J.C. Fay. *PLoS Genet* **15**: e1008375.

Malcova I, Farkasovsky M, Senohrabkova L, Vasicova P, Hasek J. 2016. New integrative modules for multicolor-protein labeling and live-cell imaging in Saccharomyces cerevisiae. *FEMS Yeast Res* **16**.

Matheson K, Parsons L, Gammie A. 2017. Whole-Genome Sequence and Variant Analysis of W303, a Widely-Used Strain of *Saccharomyces cerevisiae*. *G3 Genes|Genomes|Genetics* **7**: 2219–2226.

McLaren W, Gil L, Hunt SE, Riat HS, Ritchie GRS, Thormann A, Flicek P, Cunningham F. 2016. The Ensembl Variant Effect Predictor. *Genome Biology* **17**.

Saha D, Gregor JB, Hoda S, Eastman KE, Gutierrez-Schultz VA, Navarrete M, Wisecaver JH, Briggs SD. 2024. Candida glabrata maintains two HAP1 ohnologs, HAP1A and HAP1B, for distinct roles in ergosterol gene regulation to mediate sterol homeostasis under azole and hypoxic conditions. *mSphere* **9**: e0052424.

Song G, Dickins BJA, Demeter J, Engel S, Dunn B, Cherry JM. 2015. AGAPE (Automated Genome Analysis PipelinE) for Pan-Genome Analysis of Saccharomyces cerevisiae ed. J. Schacherer. *PLoS ONE* **10**: e0120671.

Sprouffske K, Wagner A. 2016. Growthcurver: an R package for obtaining interpretable metrics from microbial growth curves. *BMC Bioinformatics* **17**: 172.

Tange O. 2022. GNU Parallel 2022-08-22 (Rushdie’). https://zenodo.org/record/7015730 (Accessed August 28, 2025).

Wagih O, Usaj M, Baryshnikova A, VanderSluis B, Kuzmin E, Costanzo M, Myers CL, Andrews BJ, Boone CM, Parts L. 2013. SGAtools: one-stop analysis and visualization of array-based genetic interaction screens. *Nucleic Acids Research* **41**: W591–W596.

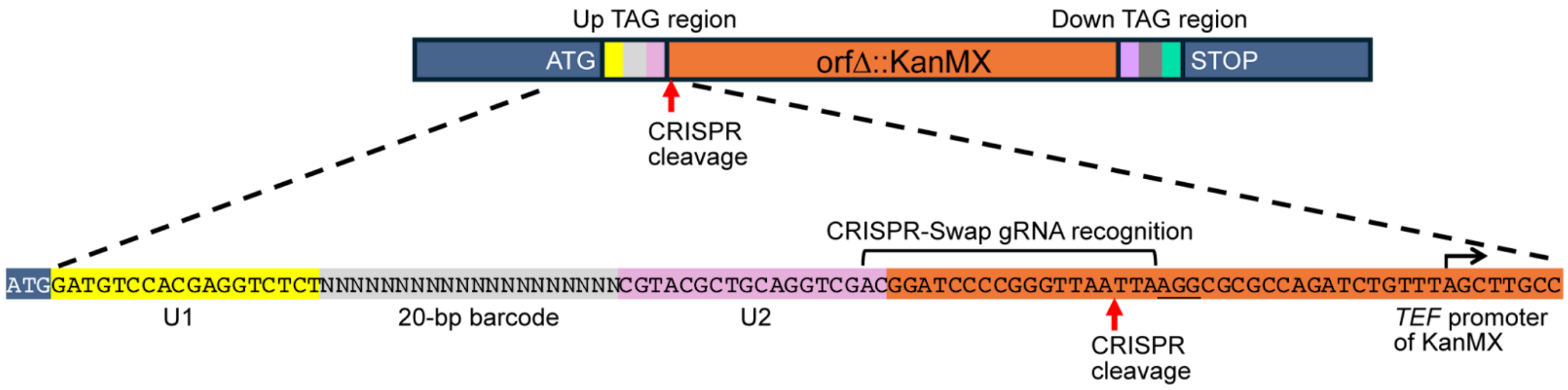

Supplemental Figure S1. The recognition site of the CRISPR-Swap guide RNA in the linker region of the KanMX cassette. The inserted KanMX cassette in each YKO strain contains 20-bp barcodes at each end that are flanked by constant regions (U1 and U2) present in all YKO strains. On the 5′ and 3’ sides of the cleavage site, there are 71 bp and 1510 bp of KanMX cassette sequence, respectively.

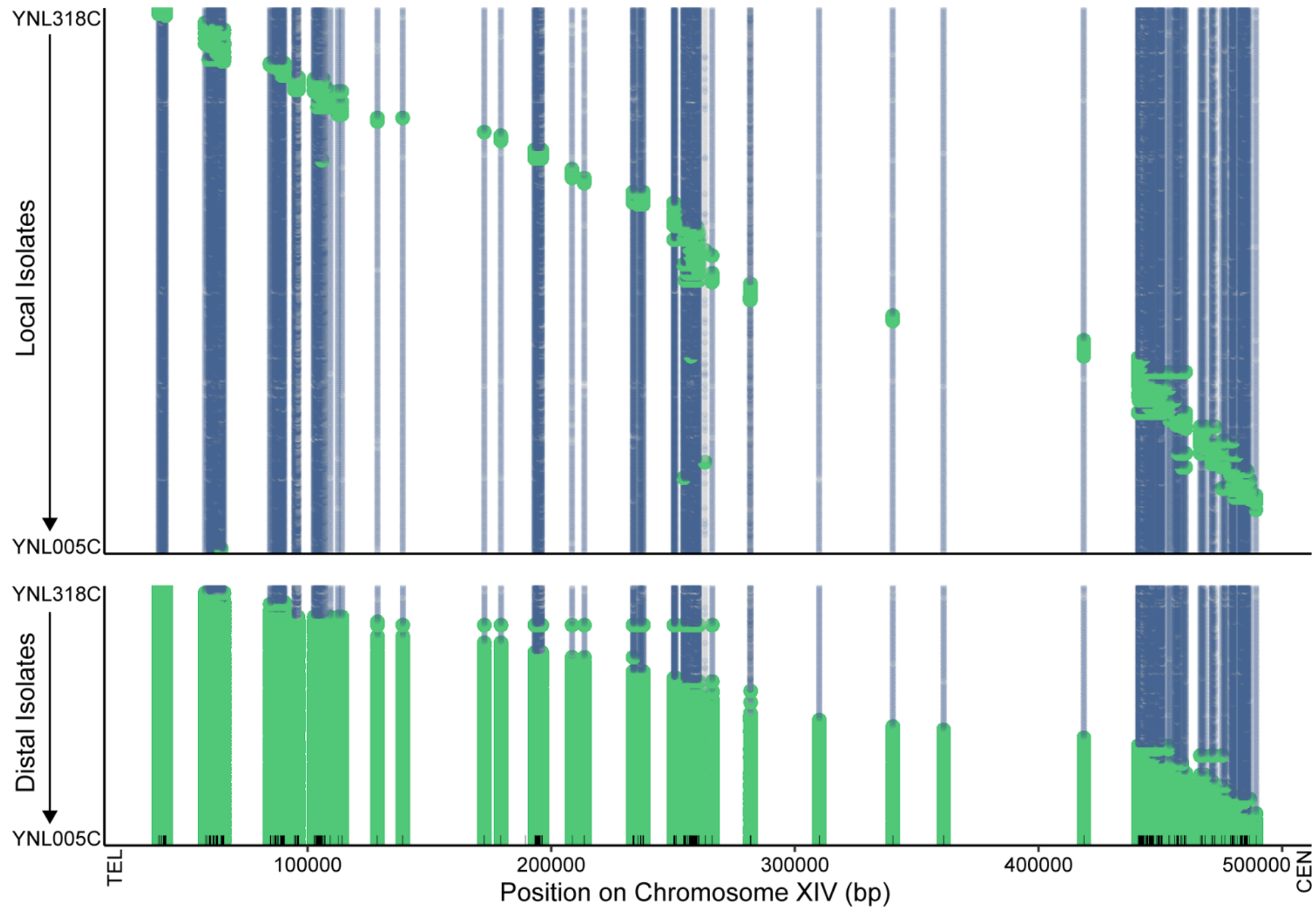

Supplemental Figure S2. Overview of all repair tracks in the CRI-SPA-Map isolates ordered by targeted ORF. W303 alleles are in green and BY alleles in blue. The gaps between alleles are caused by the clustering and sparsity of variants, marked by tick marks on the bottom of the plot. The DNA-fingerprint variant at chrXIV:189585, which segregates in the YKO collection was removed.

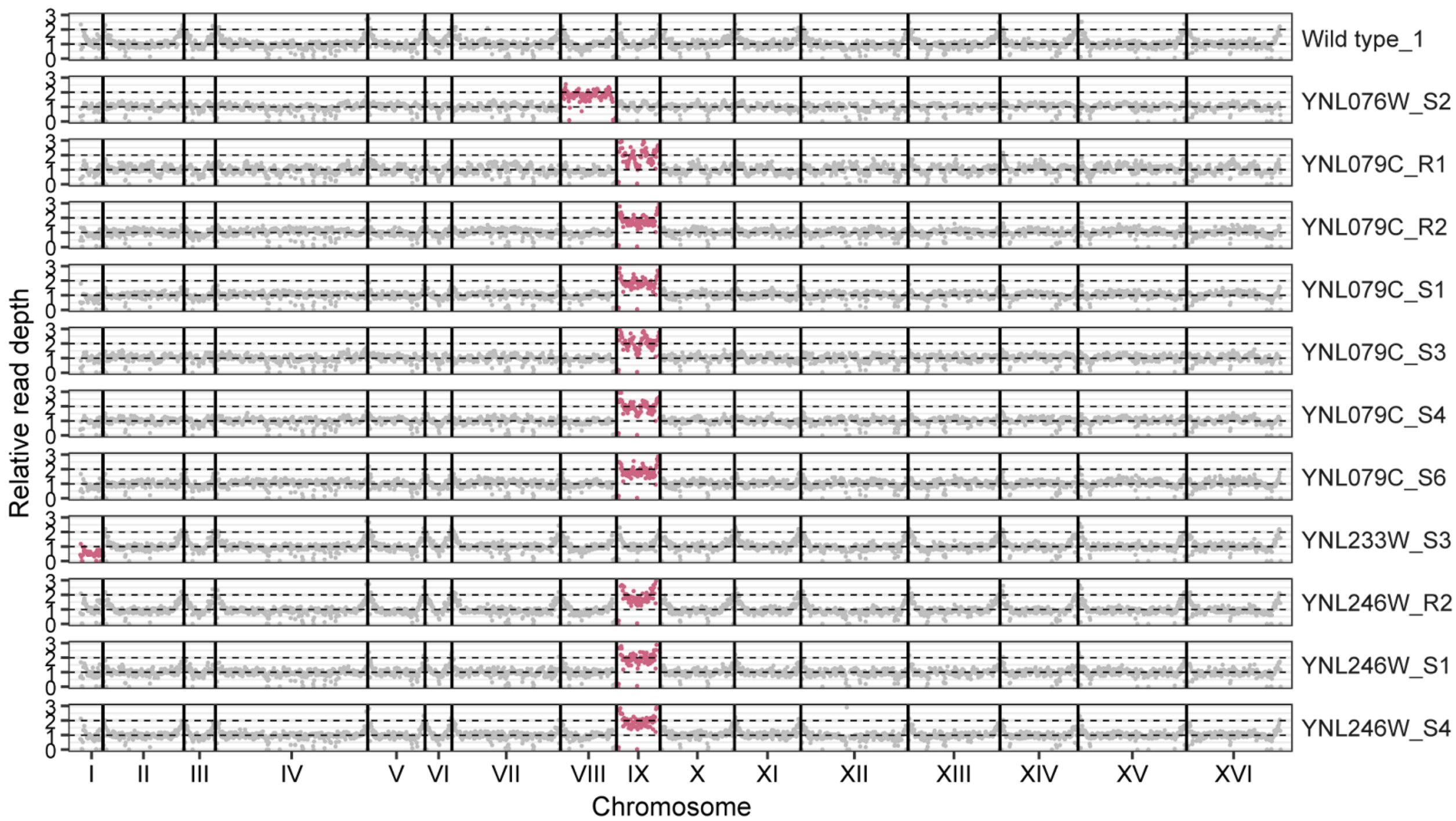

Supplemental Figure S3. Isolates with aneuploidies. Plotted is the ratio of the read depth in 5,000-bp bins compared to the average read depth across the nuclear genome for each isolate. Chromosomes with detected aneuploidies are colored in pink. Shown for comparison is the wild-type isolate 1, which has no detected aneuploidy.

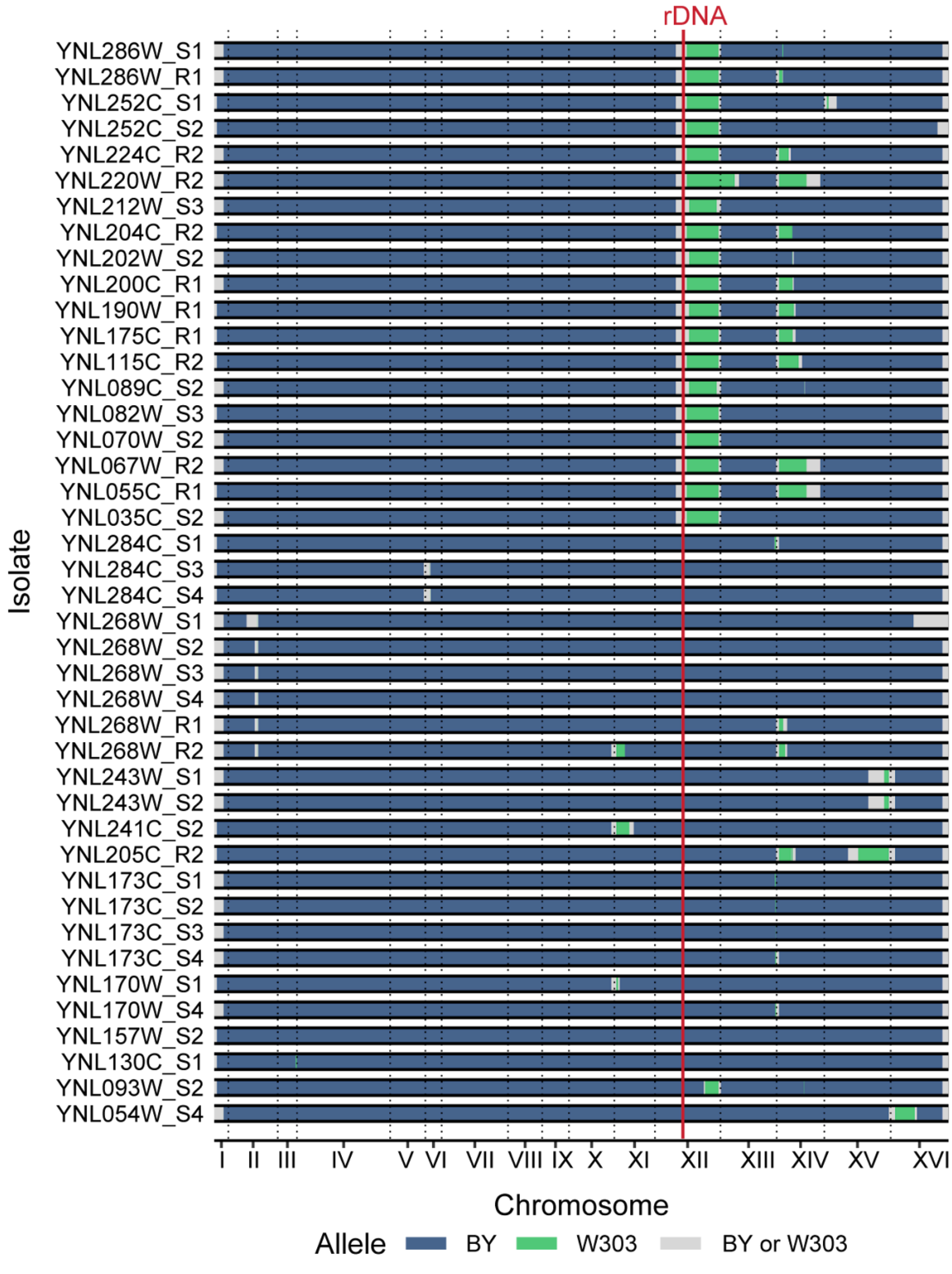

Supplemental Figure S4. Isolates with runs of W303 variants on chromosomes other than chrXIV-L.These isolates mostly come from different YKO strains.The region from the rDNA to the telomere is the most frequently observed location of W303 variant integration other than the targeted chrXIV-L. The exceptions include all six isolates of the YNL268W YKO strain that have W303 variants on chrII-L due to the presence of the *LYS2* gene in these isolates. All 4 isolates from YNL173C YKO strain (only Hyg^S isolates were recovered) have W303 variants on chrXIII-R suggesting this is the site of the YKO ORF that was inadvertently targeted.

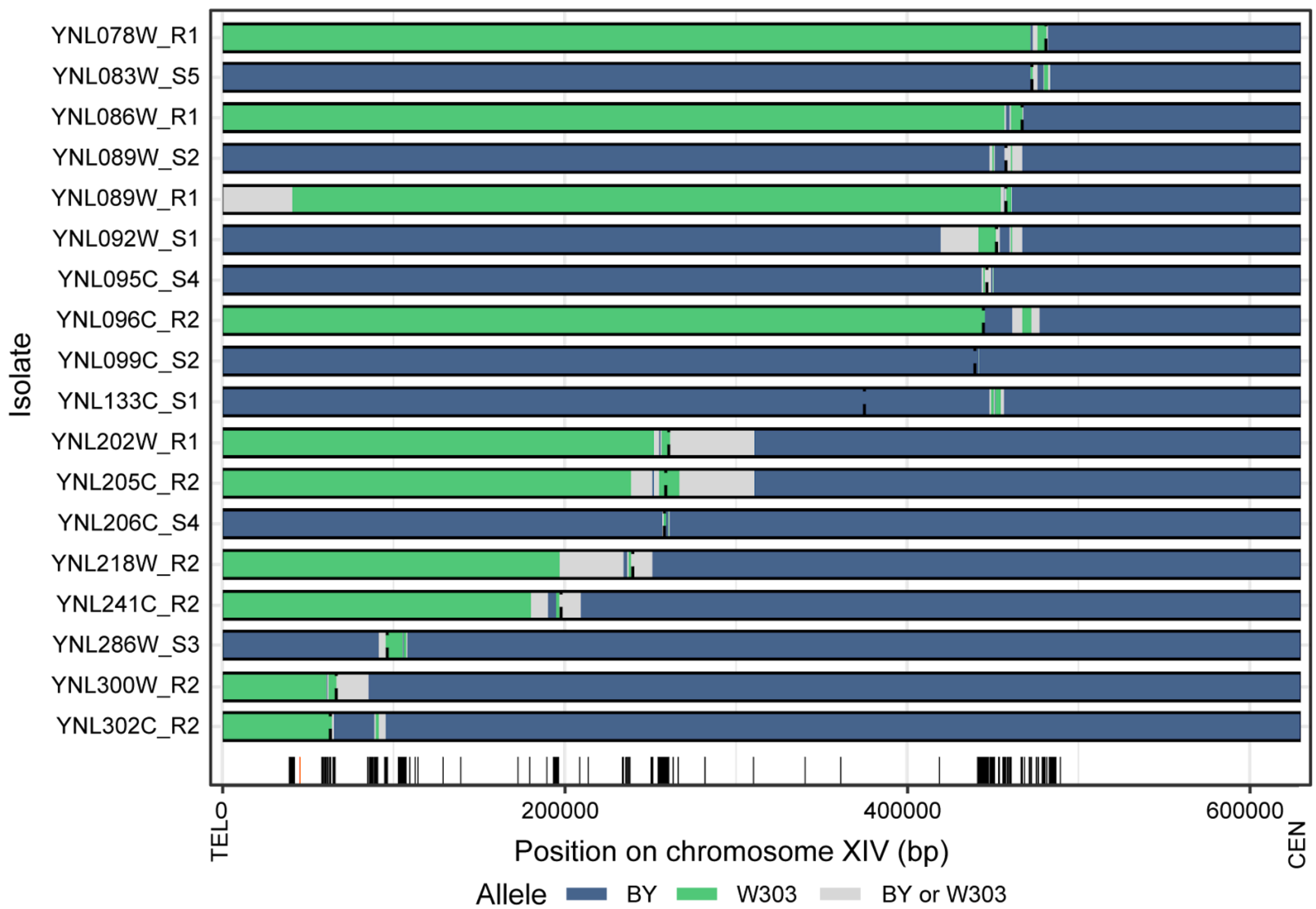

Supplemental Figure S5. Isolates with mosaic tracts of BY and W303 variants. The sequence between the last tract of variants to the next tract of variants of the opposite allele could originate from either the BY or W303 strain and is therefore designated as gray. The position of the targeted ORF is shown with a dotted line. The positions of the variants are marked by vertical lines along with the position of the telomere (TEL) and centromere (CEN) at the bottom of the plot.

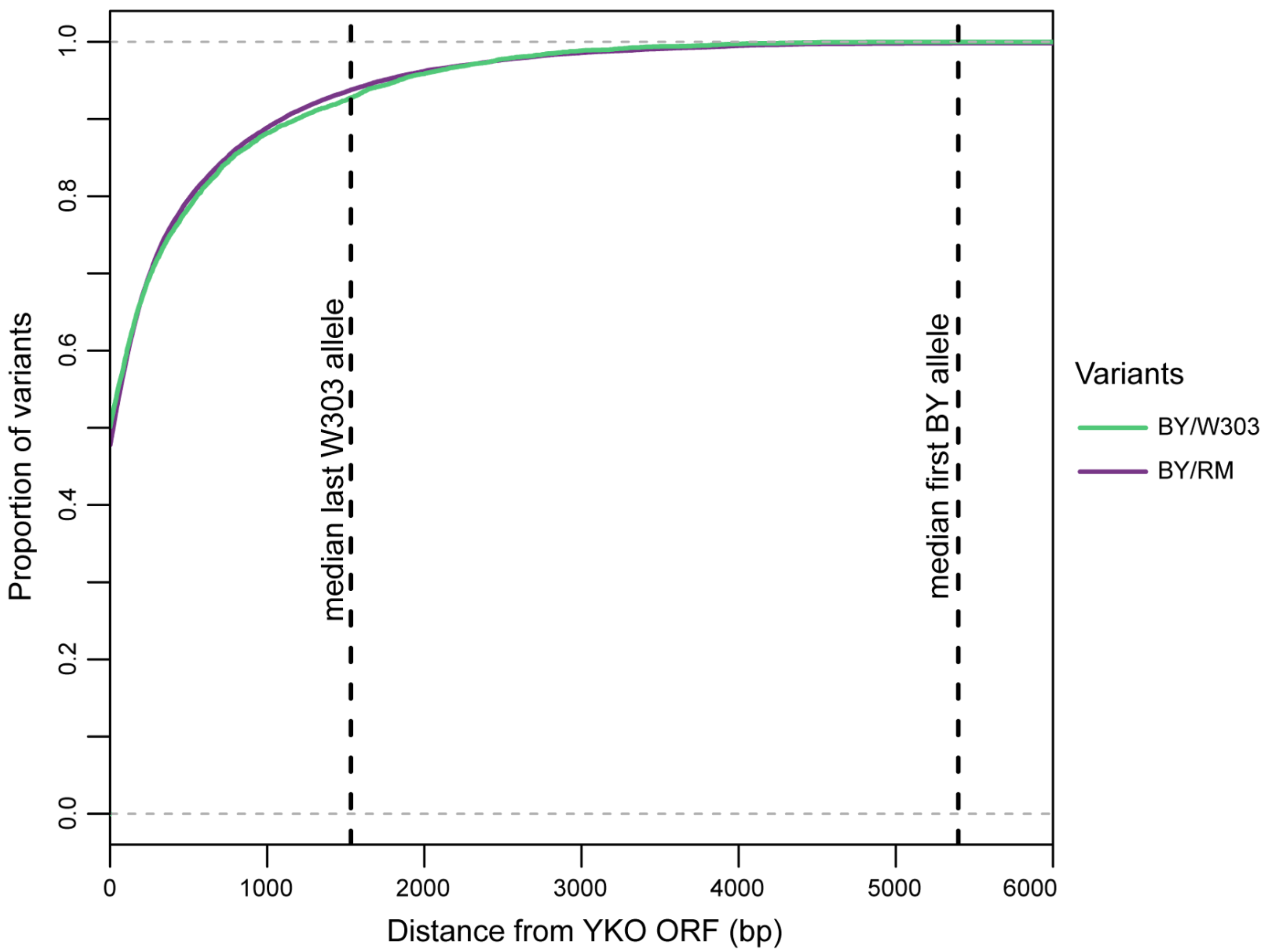

Supplemental Figure S6. The great majority of *S. cerevisiae* variants are within the typical repair tract length from an ORF in the YKO collection. In the CRI-SPA-Map isolates, the median length of W303 allele tracts at each end of the YKO ORFs is between 1,532 bp for the last W303 allele and 5,397 bp for the first BY allele. The percentage of BY/W303 variants that are located within these lower and upper median tract length boundaries are 92.8% and 99.9%, respectively. The percentage of BY/RM variants that are located within these boundaries is similar (93.8% and 99.8%).

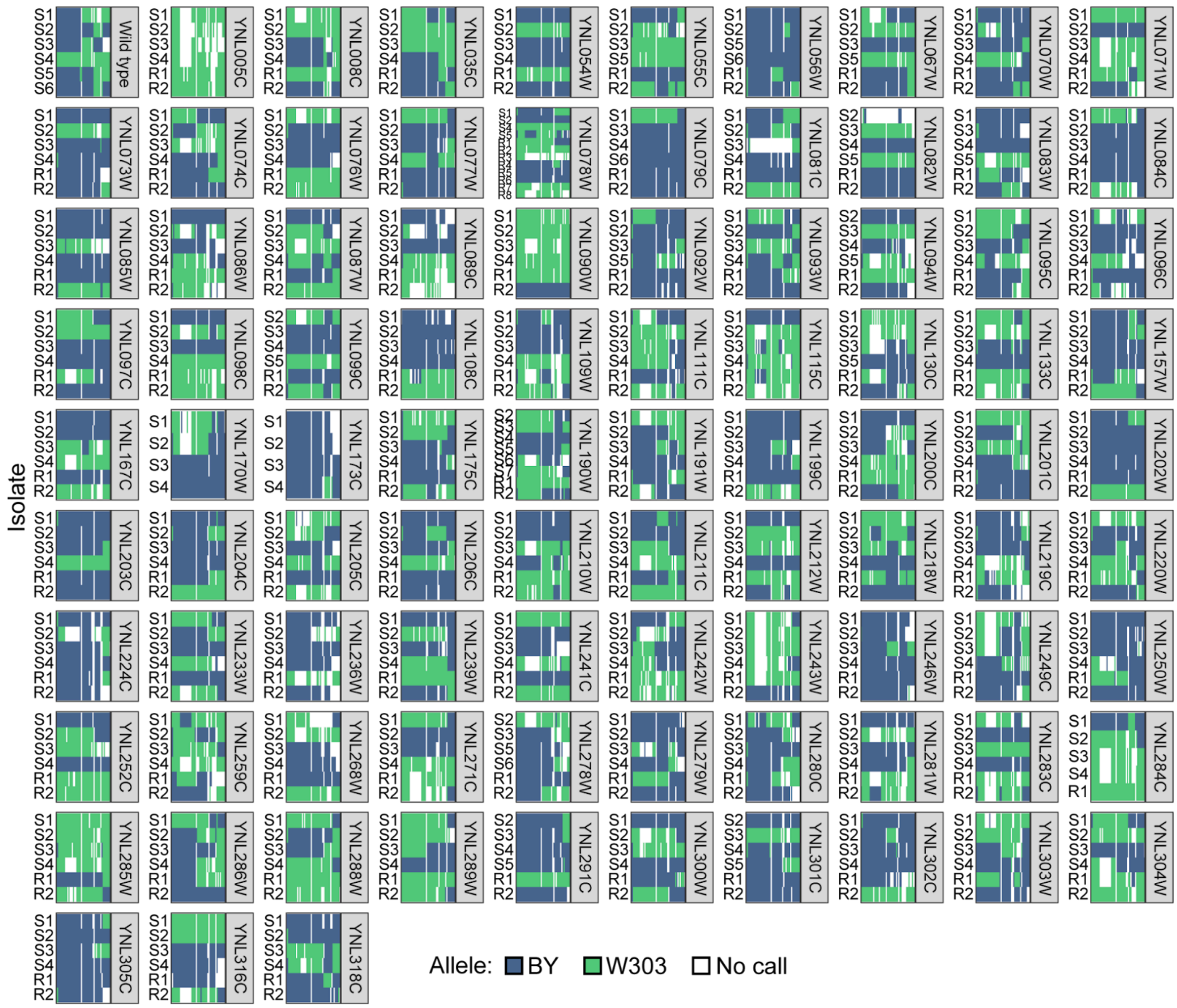

Supplemental Figure S7. Mitochondrial variants in the CRI-SPA-Map isolates. Variant calls were determined at 48 sites. Isolates from each YKO strain and the wildtype strain had mitochondria with differing genotypes, except for YNL005C. All YNL005C isolates had only W303 variant calls. YNL005C/*MRP7* encodes a subunit of the mitochondrial ribosome and deletion of this gene causes loss of mitochondria. Therefore, the potential absence of mitochondria in the YNL005C/*MRP7* YKO strain may have led to CRI-SPA-Map isolates with only W303 mitochondria.

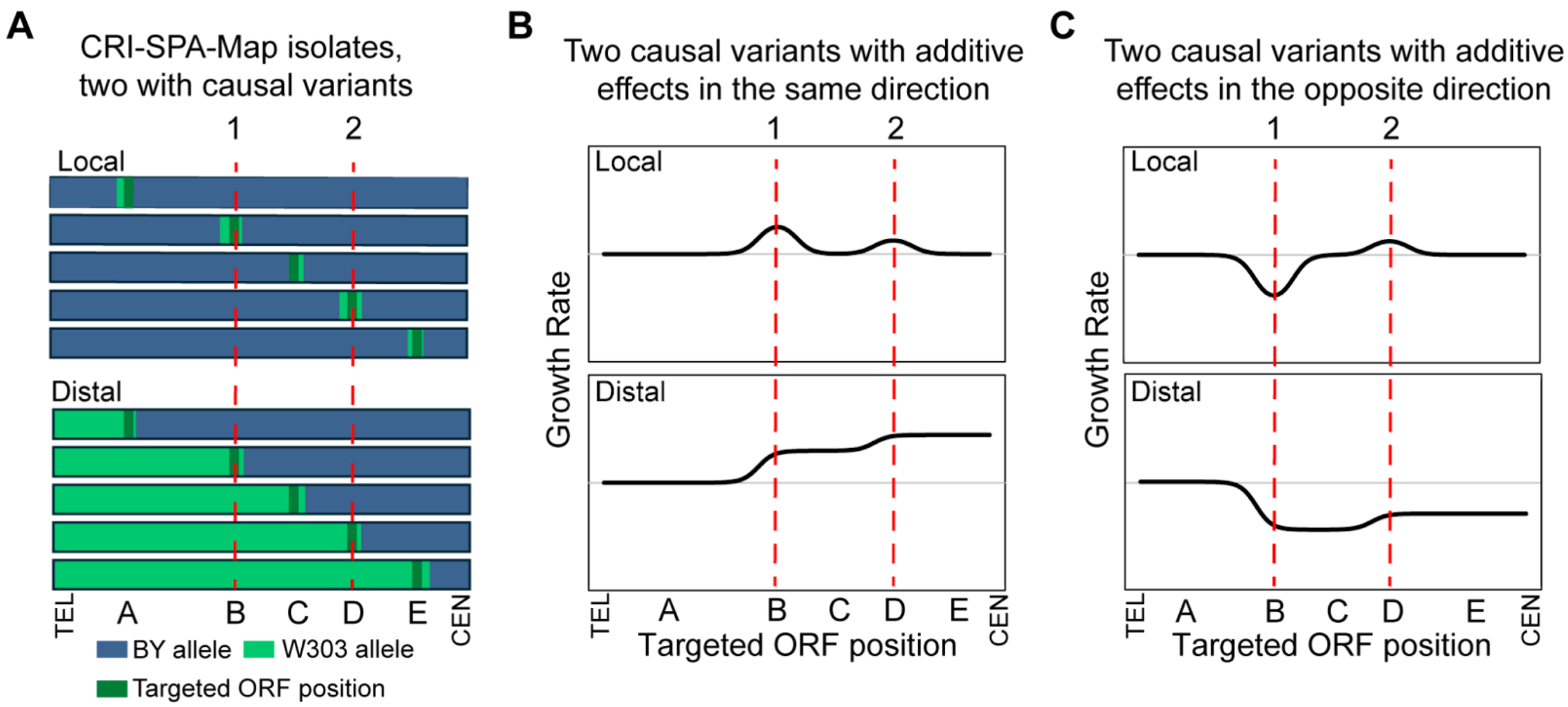

Supplemental Figure S8. Illustration of patterns expected for ORF-based mapping results using CRI-SPA-Map. (A) One local and one distal CRI-SPA-Map isolate is shown for each of five targeted ORFs, labeled A–E and marked in dark green. The incorporated W303 donor variants are shown in green; they include all variants in the targeted ORF. The BY recipient strain alleles are shown in blue. A causal variant, depicted with a red dashed line, is present in ORFs B and D. (B) Expected result of ORF-based mapping using the targeted ORFs and isolates in (A) and additional isolates from adjacent ORFs not shown, assuming both causal variants have effects in the same direction. (C) as in (B), but assuming the two variants have effects in opposite directions. In local isolates, each variant is expected to form a positive or a negative peak, depending on the direction of the effect. In the distal isolates, the effect of a causal variant will be shared by all isolates created from strains with YKO cassettes on the centromeric side of the variant. Therefore, steps at each causal variant will be evident.

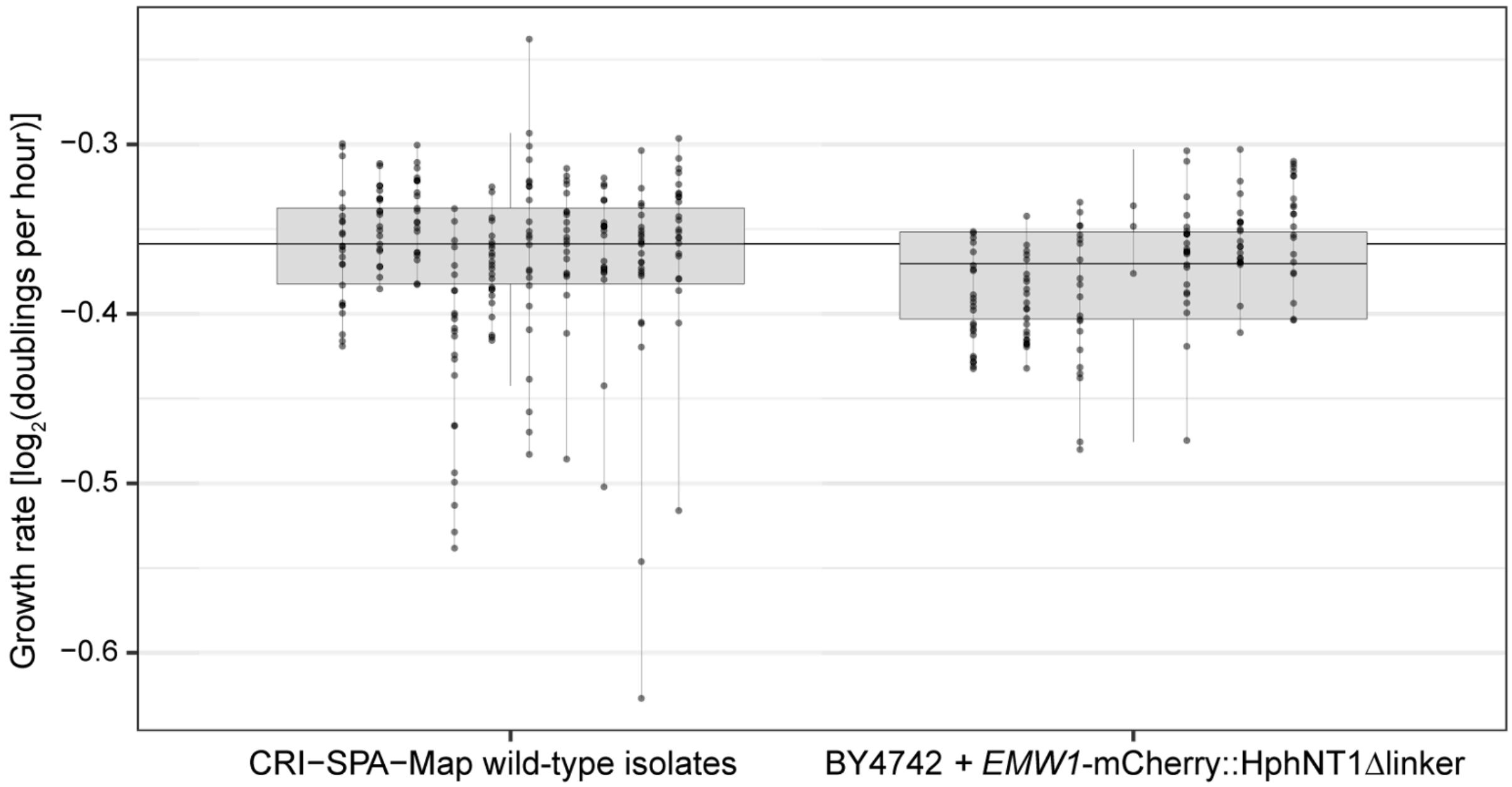

Supplemental Figure S9. The effect on growth in YPD liquid of the HphMX cassette integrated near the telomere on the left arm of chromosome XIV. The cassette did not have a significant effect on growth rate (*t*-test *p*-value = 0.47). Growth measurements from the same isolate are connected by a vertical line. Box plots designate the central 50% of the data with the median marked as a horizontal line within each box.

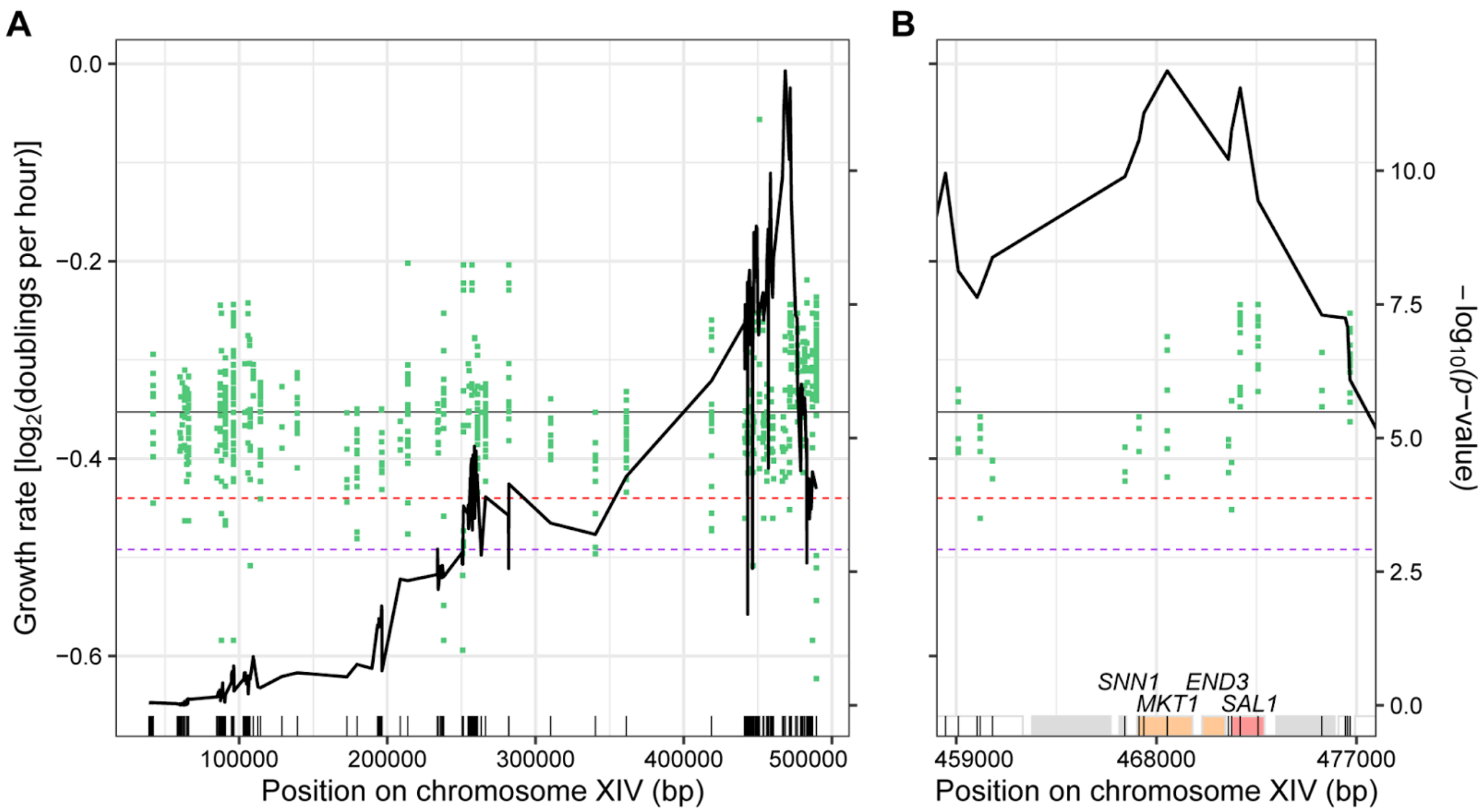

Supplemental Figure S10. Incorporation of genotype data in CRI-SPA-Map for distal isolates. (A) The variants on the left arm of chromosome XIV are depicted with vertical tick marks on the bottom. Green points depict the centromeric end of the W303 allele tracts in each of 163 distal isolates, as well as the growth rate of each isolate in liquid YPD. The solid black line shows the -log10(*p*-value) at each variant as indicated by the right y-axis in panel B. The median growth rate of all distal isolates is denoted by a horizontal gray line. Dashed lines denote significance thresholds as determined by Bonferroni correction (red) and 1,000 permutations (purple). (B) Zoom-in on the region containing the most significant variants. The ORFs in this region are depicted as boxes and those used in the ORF-based mapping are colored based on the significance of their effect on growth as in Figure 2, bottom panel.

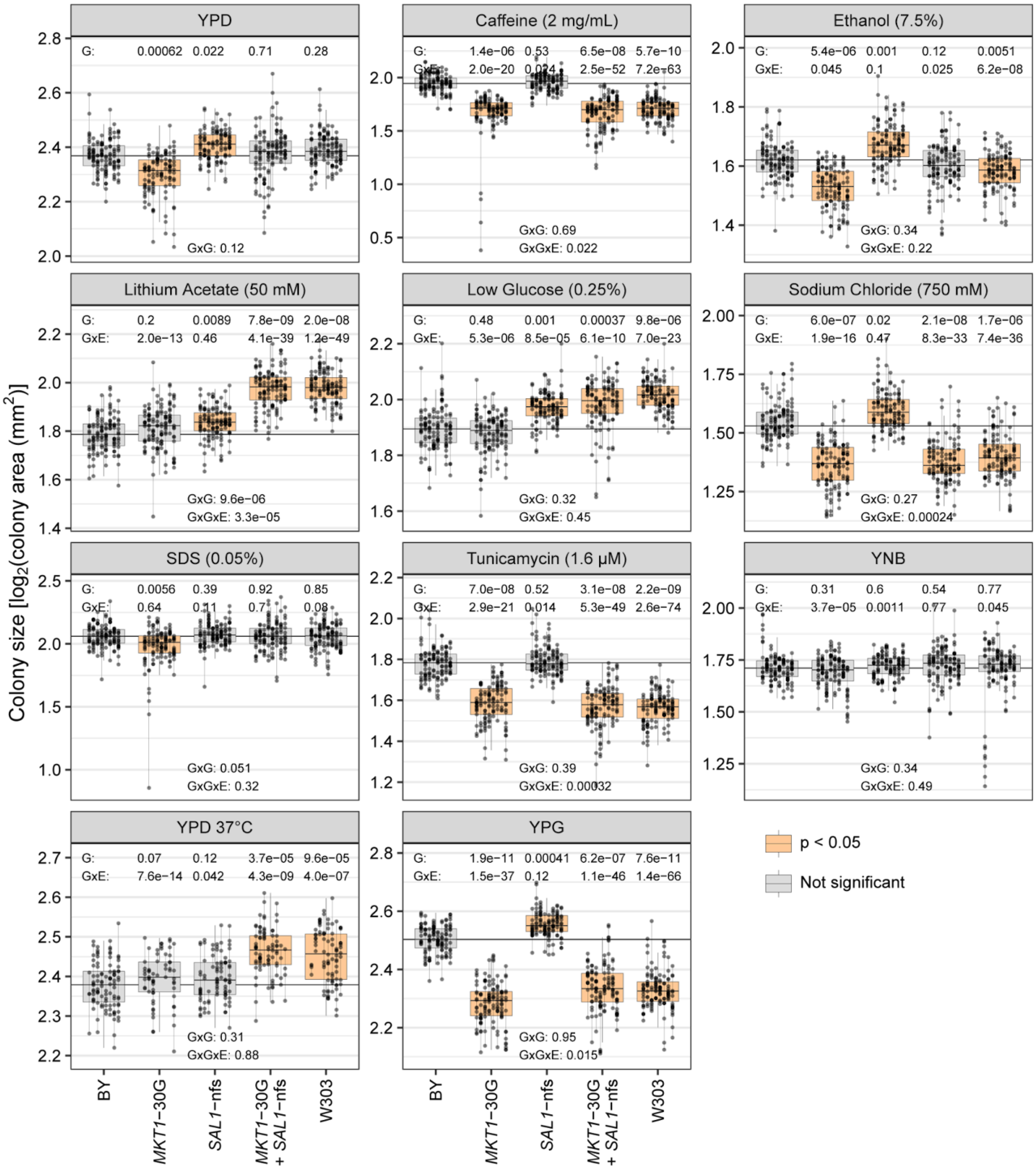

Supplemental Figure S11. Genetic and environmental interactions of *MKT1-30G* and *SAL1-nfs* alleles. Replicate growth measurements from the same strain are connected by a vertical line. Box plots designate the central 50% of the data with the median marked as a horizontal line within each box. The median of the BY wild-type strain is depicted as a horizontal line through the plot. Box plots are shaded orange if $p < 0.05$. The p-values for the various tests of genotype (G) and environmental (E) interactions are shown.

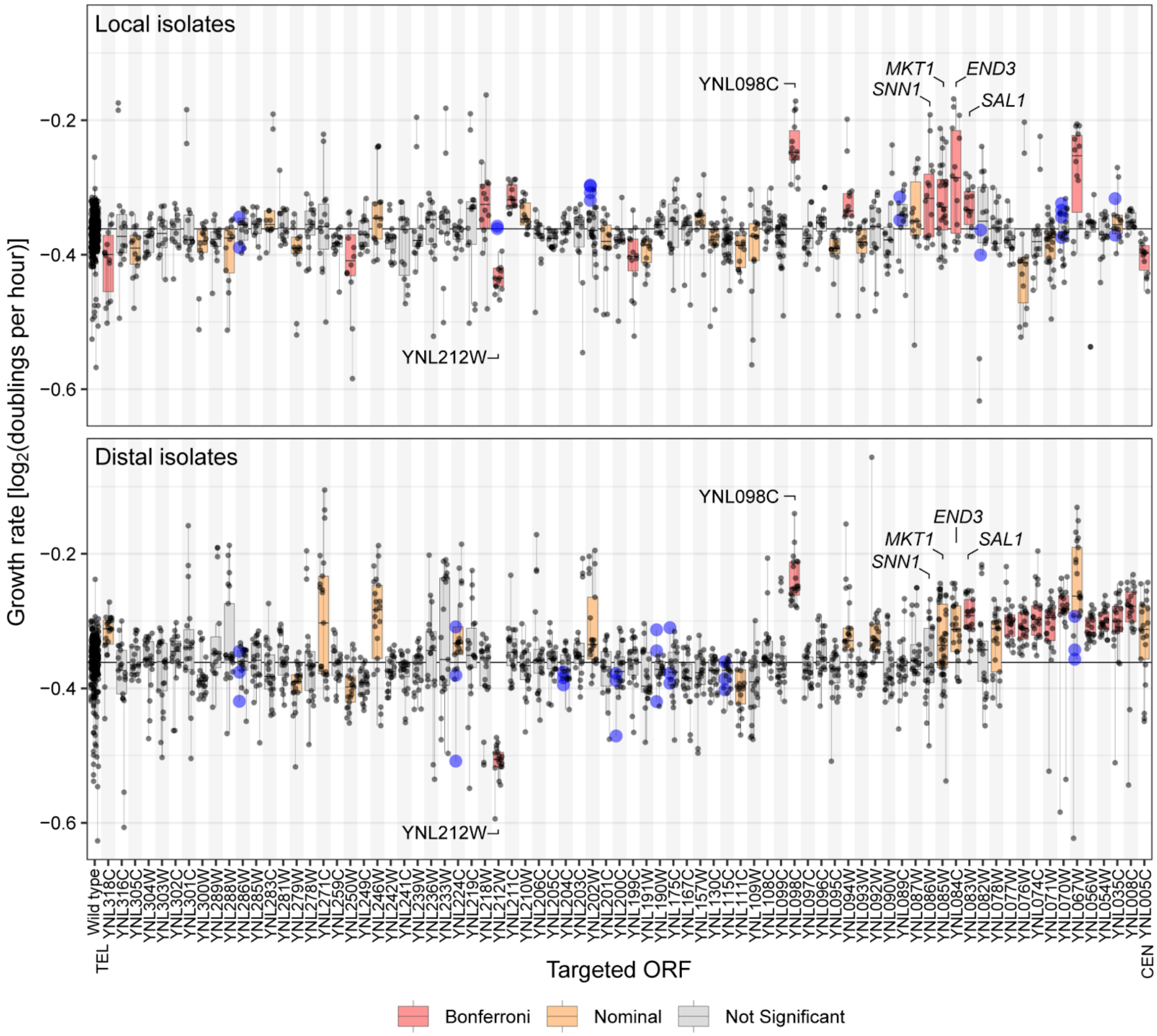

Supplemental Figure S12. Growth rate in liquid YPD of local and distal isolates including those with recombination on chrXII-R. Local isolates are in the top panel and distal isolates in the bottom panel. Vertical lines connect replicate measurements (points) for each isolate. The isolates are grouped by targeted ORFs and are ordered by position on the chromosome from telomere (TEL, left) to centromere (CEN, right). Box plots designate the central 50% of the data with the median marked as a horizontal line within each box. The median of the BY wild-type strain is depicted as a horizontal line through the plot. The blue points are measurements from isolates carrying the *HAP1* allele from W303. Growth rate is compared to the wild-type isolates shown on the far left. Red boxes signify significance at the Bonferroni threshold of $p < 0.00064$, and orange at $p < 0.05$. The results were overall very similar to those that excluded these isolates (compare to Fig. 3) with two exceptions: YNL035C rose from not significant to nominal significance in local isolates, and YNL067W dropped from Bonferroni to nominal significance in distal isolates.
