# Supplementary material for: High-resolution genotype-free mapping of genetic variation with CRI-SPA-Map
Source: Genome Res. 2026 May;36(5):1016–28. doi: 10.1101/gr.281514.125 (PMC13445715; doi:10.1101/gr.281514.125)
Supplement: Supplementary file 2 [file Supplemental_File_S1.zip › Supplemental_File_S1/Supplemental_File_S1_Legend.docx]

**Supplemental File S1.** Position of W303 tracts in 555 isolates
